# Targeted multispectral filter array design for the optimization of endoscopic cancer detection in the gastrointestinal tract

**DOI:** 10.1117/1.JBO.29.3.036005

**Published:** 2024-03-29

**Authors:** Michaela Taylor-Williams, Ran Tao, Travis W. Sawyer, Dale J. Waterhouse, Jonghee Yoon, Sarah E. Bohndiek

**Affiliations:** a University of Cambridge, Department of Physics, Cavendish Laboratory, Cambridge, United Kingdom; b University of Cambridge, Cancer Research UK Cambridge Institute, Cambridge, United Kingdom; c University of Arizona, Wyant College of Optical Sciences, Tucson, Arizona, United States; d University College London, Wellcome/EPRSC Centre for Interventional and Surgical Sciences, London, United Kingdom; e Ajou University, Department of Physics, Suwon-si, Republic of Korea

**Keywords:** endoscopy, colonoscopy, esophageal cancer, colon cancer, multispectral filter array, multispectral imaging

## Abstract

**Significance:**

Color differences between healthy and diseased tissue in the gastrointestinal (GI) tract are detected visually by clinicians during white light endoscopy; however, the earliest signs of cancer are often just a slightly different shade of pink compared to healthy tissue making it hard to detect. Improving contrast in endoscopy is important for early detection of disease in the GI tract during routine screening and surveillance.

**Aim:**

We aim to target alternative colors for imaging to improve contrast using custom multispectral filter arrays (MSFAs) that could be deployed in an endoscopic “chip-on-tip” configuration.

**Approach:**

Using an open-source toolbox, Opti-MSFA, we examined the optimal design of MSFAs for early cancer detection in the GI tract. The toolbox was first extended to use additional classification models (k-nearest neighbor, support vector machine, and spectral angle mapper). Using input spectral data from published clinical trials examining the esophagus and colon, we optimized the design of MSFAs with three to nine different bands.

**Results:**

We examined the variation of the spectral and spatial classification accuracies as a function of the number of bands. The MSFA configurations tested showed good classification accuracies when compared to the full hyperspectral data available from the clinical spectra used in these studies.

**Conclusion:**

The ability to retain good classification accuracies with a reduced number of spectral bands could enable the future deployment of multispectral imaging in an endoscopic chip-on-tip configuration using simplified MSFA hardware. Further studies using an expanded clinical dataset are needed to confirm these findings.

## 1 Introduction

Multispectral imaging (MSI) is an emerging technique that holds promise in a range of biomedical applications, from monitoring of wound healing to enhancing contrast for early cancer in endoscopy.^1^^–^^3^ MSI is based on the premise that tissues have their own spectrally unique reflectance fingerprint^4^^,^^5^ arising from optical absorption and scattering processes, which are fundamentally altered by structural and biochemical changes that occur during disease progression. In cancer, for example, aberrant angiogenesis leads to neovascularization that primarily alters optical absorption due to changes in hemoglobin (Hb) abundance and oxygenation levels. Furthermore, changes in cancer cell morphology, organelle distribution, and size, alter tissue scattering properties.^2^^,^^3^^,^^6^^–^^10^ Measurements of the spectral fingerprint of different tissue types have, therefore, been widely used to reveal the presence of cancer in excised tissue samples and *in situ* in patients.^11^^–^^14^

Clinical white light endoscopy and narrow band imaging offer limited forms of MSI, targeting three color (red, green, and blue) vision and two color (415±10 and 540±10  nm) hemoglobin absorption, respectively. MSI incorporating a larger number of spectral bands is not yet widely used in clinical applications.^15^ Key challenges in clinical translation include the design of suitable instrumentation as well as the development of appropriate expertise in operators and interpreters.^2^ In terms of hardware, MSI systems typically require a tradeoff between spectral, spatial, and temporal resolution.^5^^,^^16^^,^^17^ Broadly, spectral imaging can be implemented via four imaging system configurations: point-scanning 1D spectrometer, line-scanning 2D spectrometer, wavelength-scanning image sensor, or a snapshot imaging spectrometer.^1^^,^^11^ The latter acquires the full 3D (x, y, wavelength) MSI data cube in a single acquisition, whereas the former options require scanning of either the spatial dimension(s) or the spectral dimension. These requirements can often lead to bulky and complex hardware as well as offline data reconstruction. In endoscopy, real-time operation is required to ease operation and interpretation as well as to account for patient movement during diagnostic procedures.^11^^,^^18^^,^^19^ Although snapshot MSI systems can achieve high temporal resolution, limited only by the camera frame rate, the low optical throughput and compromise between spatial and spectral resolution can degrade image quality compared to spatial-scanning systems.

To optimize the image quality in snapshot systems, one can target the spectral properties of the system to strategically sample incoming light at particular wavelengths known to be information rich in the target application of interest. The resulting spectral reflectance fingerprint of the given disease state can then be unmixed or classified accurately with fewer spectral samples.^16^ Existing endoscopic systems use wavelength-scanning typically with a white light source and filter wheel for spectral sampling, however, this can become problematic in terms of temporal resolution and image co-registration as one increases the number of spectral samples. An alternative snapshot solution uses a multispectral filter array (MSFA) atop an imaging sensor.^17^^,^^20^^,^^21^ Using absorptive or interference techniques, the MSFA filters the spectrum of the incoming light detected on a pixel-by-pixel basis, with a mosaic of filters deposited pixel-by-pixel across the sensor.^3^^,^^22^^,^^23^ Following demosaicking and interpolation, the full image cube, including spatial and spectral information, is retrieved in a single snapshot.^24^ Targeting the spectral properties of the MSFA to a given application thus maximizes the spatial resolution^17^^,^^25^^,^^26^ and is feasible from a manufacturing perspective,^27^^,^^28^ suggesting potential to address the aforementioned clinical unmet needs of MSI.

Here, we explore the potential for optimizing spectral sampling for early cancer detection in the gastrointestinal (GI) tract. We examine two published hyperspectral datasets available from prior endoscopy clinical trials: the first focused on detecting changes in tissue spectra in dysplasia and early cancer in the esophagus and the second focused on measuring spectra from polyps and residual tissue postresection in the colon. We expand the capability of the open-source Opti-MSFA toolbox to select the bandwidths and center wavelengths for 3 to 9 filters.^17^ Moreover, we then use the capability of the Opti-MSFA toolbox to tailor the spatial properties of an MSFA to sample these spectral bands according to a synthetic input hypercube with reference endmembers. We set parameters for the spectral properties of the filters using a merit function that represents the resulting error that would occur in sampling the spectra with the designed system.^17^ Our results, derived using a range of merit functions, indicate the importance of end-to-end optimization for customizing filters using appropriate merit functions when designing MSFAs. We demonstrate that customized spectral filters can be efficiently designed and optimized to detect GI cancers using the Opti-MSFA toolbox based on the chosen datasets. These promising results suggest targeted design of MSFAs could provide similar MSI performance as full hyperspectral imaging, obviating the need for complex hyperspectral hardware in endoscopic applications.

## 2 Methods

The MSFA design exploited hyperspectral data collected during endoscopies of the esophagus and colon *in vivo* (Sec. 2.1),^8^^,^^9^ which was then analyzed using the Opti-MSFA toolbox (Sec. 2.3)^17^ to optimize the filters. Different classification techniques and unmixing functions were incorporated into the Opti-MSFA toolbox to develop the optimal MSFA for detecting cancers in these organs (Secs. 2.4–2.6). Additionally, a merit function based on the unmixing of oxy- and deoxyhemoglobin was used for comparison (Sec. 2.7).

### 2.1 Hyperspectral Datasets of Esophageal and Colon Tissues

**Fig. 1:**
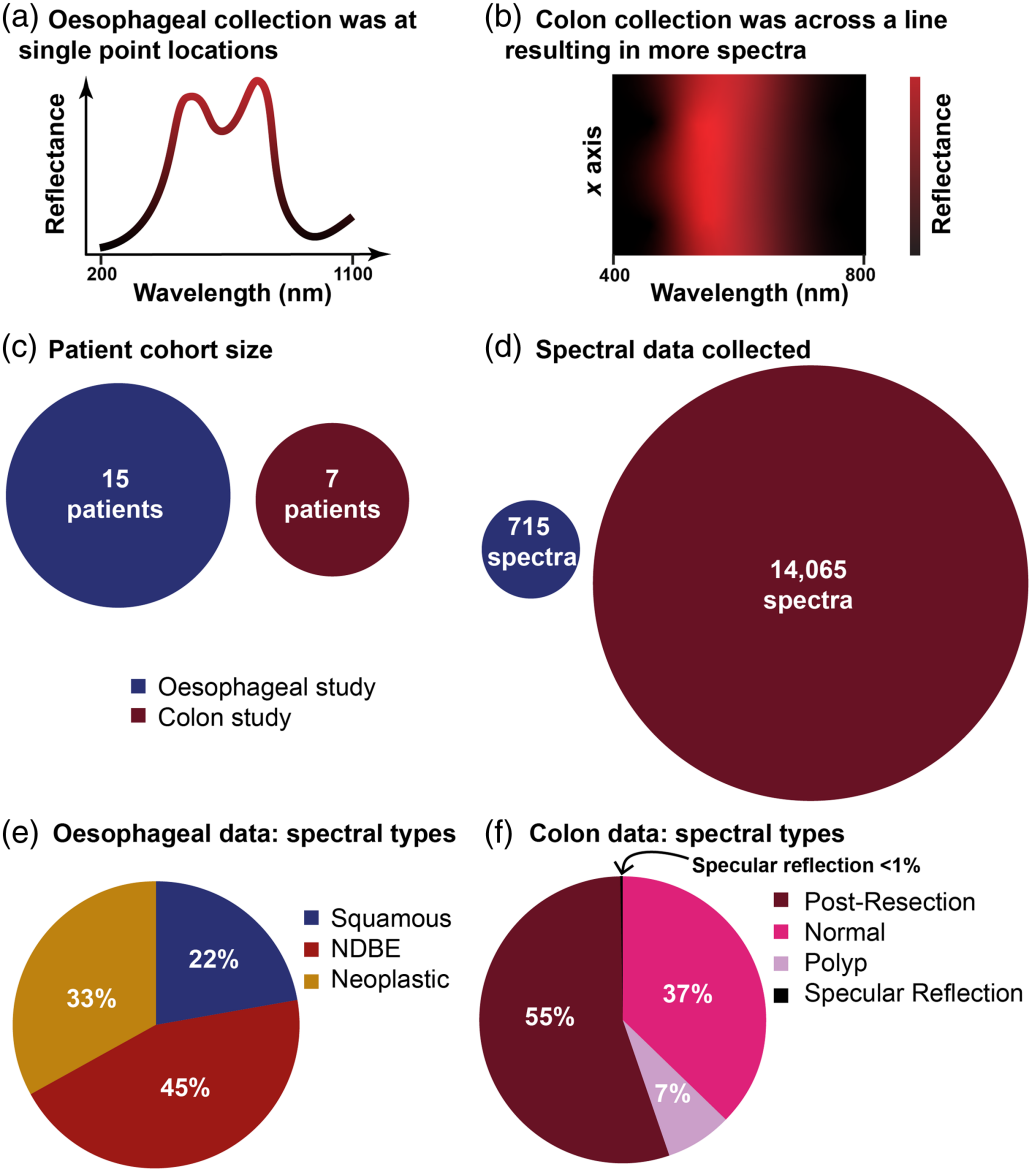
Overview of the clinical datasets. Illustration of the data collection method for the (a) esophageal and (b) colon study. (c) Patient cohort size and (d) total spectra for each of the studies are compared to illustrate how the line scanning method results in significantly more data. (e) Esophageal and (f) colon spectra are well-balanced between disease types.

Hyperspectral data (Fig. 1) were collected in two prior clinical studies of hyperspectral endoscopy,^8^^,^^9^ the methods for which are briefly summarized below. Both datasets were collected using a “babyscope” (PolyScope and Polydiagnost) that can be threaded through the accessory channel of a clinical gastroscope or colonoscope.

For the esophageal study,^8^ the trial was reviewed by the Cambridgeshire Research Ethics Committee and was approved in March 2018 (18/NW/0134) and registered at ClinicalTrials.gov (NCT03388047). A broadband supercontinuum light source was used for illumination (SuperK COMPACT, NKT Photonics). The 10,000 fiberlet-imaging bundle (PD-PS-0095, PolyDiagnost) was imaged using an objective lens, and then the measured signal was split into two arms using a beam splitter. The split images were measured via a standard color camera (Grasshopper 3.0, FLIR) and spectrometer (AvaSpec-ULS2048, Avantes; spectral range 200 to 1100 nm, grating 300  lines/mm, slit size 50 mm,) to capture a structural image and averaged spectral information, respectively. Tissue spectra were collected in the esophagus from three different tissue types, determined by histopathology: healthy squamous, nondysplasia Barrett’s esophagus (NDBE), and neoplasia. These 715 spectra were collected from 15 different patients: 159 were from squamous regions; 320 from NDBE; and 236 from neoplastic regions.

For the colon study,^9^ the trial was reviewed by the OHSU Institutional Review Board (IRB18947) and registered at ClinicalTrials.gov (NCT04172493). The white light provided by the standard-of-care colonoscope was used for illumination (Olympus CF-H290). In the colon study, a 2D spectrograph (IsoPlane 160, Princeton Instruments; spectral range 400 to 800 nm, grating 150  lines/mm) was used with an electron-multiplied CCD camera (ProEM 1024, Princeton Instruments) to collect the spectral data using a line-scanning hyperspectral imaging method.^29^ Tissue spectra were collected in the colon from three different tissue types determined by the colonoscopist: normal mucosa, polyp, and postresection tissue (after polyp removal). In addition, spectral profiles of specular reflections were also captured. 14,065 spectra from 7 different patients were collected; 5269 spectra from normal regions; 1045 from polyps, 7745 postpolyp resection, and 6 were a result of specular reflection.

Both spectral datasets used in the toolbox were downsampled using interpolation to have a spectral resolution of 1 nm, to simplify the computational process, and the wavelengths were restricted to between 470 and 720 nm, which eliminated spectral regions with high noise arising from insufficient illumination power.

### 2.2 Hypercube Generation

To run the optimization, synthetic hypercubes were created (Fig. S1 in the Supplementary Material) that were then input into the Opti-MSFA toolbox to optimize the filters. The hypercubes creation process differed slightly according to the different datasets (colon and esophageal) and algorithms used for merit functions (if a training data split was required); nonetheless, the same three classification methods were applied to the two different hypercubes.

#### 2.2.1 Splitting of data

For the hypercubes used with k-nearest neighbor (kNN) and support vector machines (SVM) classification models, the dataset was split in a 4:1 ratio for the training and testing [Figs. S1(a) and S1(b) in the Supplementary Material]. For spectral angle mapping and spectral unmixing, training is not required and the methods rely on identification of a reference spectrum, hence a training/testing split of the data was not required.

#### 2.2.2 Esophageal hypercube layout

For the esophageal data, raw spectra were randomly subsampled (using a random number generator in Python) and allocated into regions in a 2×2 arrangement within an overall 80×80 shape [Figs. S1(c) and S1(d) in the Supplementary Material]. Raw spectra were selected randomly from either the testing set (kNN and SVM merit functions) or the full dataset (all other merit functions). The spatial arrangement of concentric circles for spectra from different disease types in the synthetic hypercube was designed to mirror the clinical situation where the detection of dysplasia within a background of nondysplastic Barrett’s esophageal tissue is a key challenge; concentric circles containing dysplasia within NDBE within squamous tissue were created. The esophageal hypercube was designed so that the ratio of the different tissue types in the hypercube approximated those of the underlying dataset, thus the resulting accuracies are weighted accordingly to address class imbalance.

#### 2.2.3 Colon hypercube layout

For the colon data, 14,065 tissue spectra were randomly subsampled and placed in two distinct circular regions to represent the polyp and postresection tissue surrounded by normal backgrounds, with overlapping specular reflections, again mirroring the clinical situation where polyps exist within a background of normal tissue. The colon hypercube created had a 96×96 shape with a spatial correlation of 2×2 regions, similar to the esophageal hypercube. The shape was slightly larger than the esophageal hypercube to allow for four spectral types instead of three, and the more complex spatial layout. For the unmixing hypercube, the same shape was used but size increased to 196×196 with a spatial correlation of 8×8 regions, since unmixing is more sensitive to discontinuities that occur at the boundaries due to the random generation of the hypercube.

#### 2.2.4 Input of the hypercubes into the toolbox

The edges of the hypercubes after the classification were cropped, such that the esophageal had a 70×70 shape and the colon had an 86×86 shape for classification or 186×186 shape for unmixing. This was to exclude misclassification due to the spatial demosaicking process at the edges of the MSFA sampling.

### 2.3 Opti-MSFA Toolbox

**Fig. 2:**
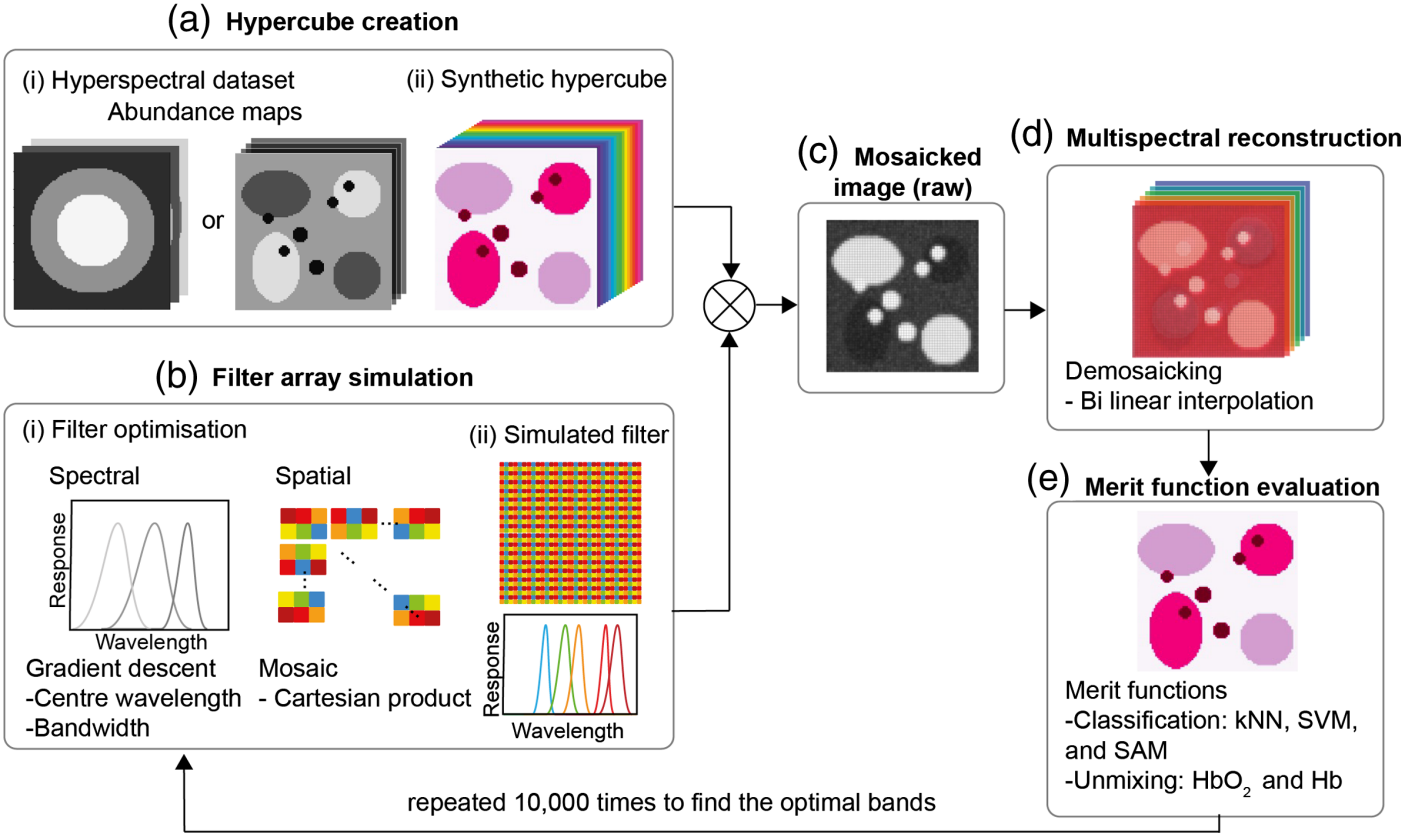
Opti-MSFA toolbox recreates the classification and unmixing process after imaging tissue using the MSFA. (a) A hypercube is created by inputting (i) spectral datasets and their abundance maps to create (ii) a synthetic hypercube. Based on this hypercube, (b) a simulated filter array and (c) a raw mosaicked image are then simulated. (d) The mosaicked image is then reconstructed using bilinear interpolation. (e) Different classification and unmixing techniques were used for endmember reconstruction, and these were used as merit functions to optimize the (b, i) spectral and spatial properties of possible filters that are then (b, ii) simulated and tested.

The open-source Python-based toolbox Opti-MSFA^17^ was used to calculate optimal spectral and spatial filter properties. Input synthetic hypercubes were composed of endmember spectra (the spectral reflectance fingerprint of a given tissue type in this case) arranged in a suitable spatial pattern [Fig. 2(a)] as outlined in Sec. 2.2. Filter array simulation was then performed [Fig. 2(b)]. Classification and unmixing-based merit functions were assessed using a gradient descent method to determine the optimal center wavelength and bandwidth, defined as the full-width at half-maximum (FWHM). The spectral optimization loop was run five times using different starting wavelengths to find five different candidate sets of spectral bands. The five different starting wavelength sets were selected by randomly sampling 10,000 candidate sets. 10,000 provided a sufficiently wide candidate set while not trying each option exhaustively and slowing down computation. The five filter sets with the highest classification accuracy in the initial tests were then refined using gradient descent. Allowed band centers were from 450 to 700 nm in 1 nm increments. Allowed FWHMs were from 10 to 30 nm in 2 nm increments to mirror the performance of fabricated MSFA. All possible spectral optimization results in the five filter sets were similar, and the final spectral properties were taken as those with the highest classification accuracy. The filters with the highest classification accuracy were usually repeated within the five sets, especially when the number of filters was small (less than five bands), resulting in similar accuracies for the five sets. Nonmatching filter sets typically implied that the gradient descent had converged on a local maximum rather than the desired global maximum.

Once the spectral bands were determined, they were then used to calculate the optimal spatial layout of the filters by an exhaustive search of all the possible spatial layouts. The possible spatial layouts are calculated by finding the Cartesian product of the different spectral bands, which effectively iterates all of the possible combinations of the filter bands:

$$B(b_{1},\ldots b_{n}{)}^{\left(i*j\right)},$$ (1)

where B are the n spectral bands $b_{1}$ to $b_{n}$, and i and j are the dimensions of the mosaic in question. The pattern that had the best classification accuracy following demosaicking [Figs. 2(c) and 2(d)] using a linear interpolation function was selected as the final optimized layout and the classification accuracy noted for comparison. Finally, the desired merit function was calculated [Fig. 2(e)]. The entire process was repeated for n=3 to 9 bands to determine optimized center wavelengths, FWHMs and MSFA layouts for different MSI configurations.

### 2.4 *k* Nearest Neighbors as a Classification Model

The kNN algorithm is a supervised classification approach that classifies an image pixel by comparing its n-dimensional spectrum to the k closest n-dimensional endmember spectra in a labeled training set, where n is the number of spectral bands chosen for optimization in Opti-MSFA. A given pixel is classified based on the consensus class of these k nearest neighboring points.^30^ kNN is relatively simple, flexible, and compatible with small to medium-sized datasets, making it ideal for use on both the esophageal and colon datasets. In addition, it previously showed initial promising results when tested on the esophageal dataset.^16^ A fivefold cross-validation was performed and the spectral region from 470 to 720 nm was assessed to determine the optimal k. The data were then shuffled to account for different combinations due to high patient variability in the underlying datasets. The process was repeated 20 times to account for the random shuffling of the datasets. Subsequently, the 20 repetitions were averaged, and it was found that k=5 gave the highest accuracy.

For optimization of the center wavelengths and FWHMs, the gradient descent algorithm was deployed. Endmember spectra were simulated by propagating the “ground truth” spectra through the simulated filters using the individual spectral data that formed part of the testing dataset. The function of merit was the accuracy of kNN classification for unmixing these simulated endmember spectra, where the kNN was trained on the training dataset.

Once the optimum spectral filter set was determined, the spatial layout of filters was optimized. The different possible arrangements of the filters in a mosaic pattern were tested exhaustively in a fivefold cross-validation process to find the layout with the highest classification accuracy. For each MSFA layout, imaging of the hypercubes was simulated using the testing dataset, which was then classified using the kNN algorithm. This was repeated four more times, so the spatial optimization was found five times on five hypercubes, and the classification accuracy was extracted for each variation. The overall classification accuracy was taken as the average classification accuracy across all five variations. The layout with the highest overall classification accuracy was chosen as the final optimized layout.

### 2.5 Support Vector Machines as a Classification Model

An alternate merit function using SVM classification was also implemented. SVM classifies data by defining hyperplanes to distinguish the data in n-dimensional space, where n is the number of bands in the filters.^5^^,^^31^ SVM is a classification method that is suited to spectral datasets due to their high dimensionality with multiple spectral bands. It can also be tuned to improve performance and parallelized to improve efficiency. Similar to kNN, SVM can be used on small to moderately sized datasets and was explored previously for used on the esophageal dataset.^16^ The SVM classification parameters for the datasets were optimized in the previous work by Waterhouse et al.,^8^ where it was found that a radial basis function with C=1000 was best for performing SVM classification. Using the Scikit-learn toolbox available in Python 3, the Gaussian radial basis function kernel was used to define the hyperplane to approximate the different class distinctions. A mid-range value of C=1000, which effectively penalizes the function for misclassification, was again found to optimize classification when assessing the ground truth spectra using the individual spectra collected. The resulting classification accuracy was used as the merit function for SVM analysis of esophageal and colon tissue.

SVM classification was implemented as a merit function. As for kNN, the classification accuracy of the filters was then tested on five simulated hypercubes in a fivefold cross-validation process. The dataset was split into a testing and training dataset in a 1:4 ratio, where the SVM algorithm was trained on training data and then tested on the hypercube made from the testing data (as outlined in Sec. 2.2). This was repeated a total of five times, so the average classification accuracy of the five hypercubes was found, and the layout with the highest classification accuracy was chosen as the optimal mosaic pattern.

### 2.6 Spectral Angle Mapping as a Classification Model

In prior analysis of the colon dataset, it was noted that the collected data exhibited low variation in the spectral signatures of each class, which lends itself to using SAM classification.^9^ With SAM, the spectra are represented as vectors and the angle between two vectors is calculated. Spectral classification is achieved by finding the reference spectrum that forms the smallest angle with the spectrum of interest by calculating the inner product using vector arithmetic^5^^,^^32^

$$\theta (\overset{\to }{t},\overset{\to }{r})=\cos^{-1}(\frac{\sum_{i=1}^{n}t_{i}r_{i}}{\sqrt{\left(\sum_{i=1}^{n}t_{i}^{2}\right)}\cdot \sqrt{\left(\sum_{i=1}^{n}r_{i}^{2}\right)}}),$$ (2)

where $\vec{t}$ and $\vec{r}$ are vectors that represent the spectrum of interest and the reference spectrum, respectively. For SAM, the classification accuracy is calculated as a ratio of the correctly classified spectra to the overall number of spectra classified. The spectral classification is assigned based on the tissue type with the lowest angle. SAM classification accuracy of 1 indicates that all testing data were correctly identified using SAM and 0 indicates that no testing data were identified correctly. SAM classification accuracy was tested as a merit function to optimize the spectral and spatial properties of the filters for 3 to 9 bands. Allowed band centers were from 470 to 700 nm in 1 nm increments. As for above, the classification accuracy of the filters was then trained and tested on five simulated hypercubes in a fivefold cross-validation process. The resulting classification accuracy was used as the merit function for SAM analysis of esophageal and colon tissue.

### 2.7 Least Squares Spectral Unmixing of Hemoglobins

In addition to classification methods, spectral data can also be subjected to linear spectral unmixing given prior knowledge of the main endmembers that contribute to the signal. In the context of biomedical tissue, oxyhemoglobin (${\mathrm{HbO}}_{2}$) and deoxyhemoglobin (Hb) are major optical absorbers that dominate optical absorption in the GI tract. Scattering is also present and can be modeled. The reflectance spectra at a given point can be described by

$$R_{r}(\lambda )=c_{\mathrm{Hb}}R_{\mathrm{Hb}}(\lambda )+c_{{\mathrm{HbO}}_{2}}R_{{\mathrm{HbO}}_{2}}(\lambda )+c_{H_{2}O}R_{H_{2}O}(\lambda )+c_{\text{scattering}}R_{\text{scattering}}(\lambda )+c_{\text{noise}},$$ (3)

where $R_{r}$, $R_{\mathrm{Hb}}$, $R_{{\mathrm{HbO}}_{2}}$, $R_{H_{2}O}$, and $R_{\text{scattering}}$ are the reflectance spectra of the pixel of interest r, for Hb, ${\mathrm{HbO}}_{2}$, $H_{2}O$, and scattering, respectively.^29^ The concentrations of deoxy- ($c_{\mathrm{Hb}}$) and oxyhemoglobin ($c_{{\mathrm{HbO}}_{2}}$) molecules, water ($c_{H_{2}O}$), and scattering ($c_{\text{scattering}}$), noise present in the imaging system or tissue ($c_{\text{noise}}$) all contribute to the overall measured spectrum $R_{r}$. The reflectance spectra can be modelled as $R_{t}$ where $c_{\mathrm{Hb}}^{\prime }$, $c_{{\mathrm{HbO}}_{2}}^{\prime }$, and $c_{\text{scattering}}^{\prime }$ are the estimated concentrations of deoxyhemoglobin, oxyhemoglobin, and scattering, respectively; in addition to an offset that accounts for noise and other errors $c_{\text{offset}}^{\prime }$:

$$R_{t}(\lambda )=c_{\mathrm{Hb}}^{\prime }R_{\mathrm{Hb}}(\lambda )+c_{{\mathrm{HbO}}_{2}}^{\prime }R_{{\mathrm{HbO}}_{2}}(\lambda )+c_{H_{2}O}^{\prime }R_{H_{2}O}^{\prime }(\lambda )+c_{\text{scattering}}^{\prime }R_{\text{scattering}}(\lambda )+c_{\text{offset}}^{\prime }.$$ (4)

Fitting Eq. (4) via a least-squares algorithm^33^ optimizes the concentration of deoxy-($c_{\mathrm{Hb}}^{\prime }$) and oxy-($c_{{\mathrm{HbO}}_{2}}^{\prime }$) hemoglobin, water ($c_{H_{2}O}^{\prime }$), scattering ($c_{\text{scattering}}^{\prime }$), and the offset ($c_{\text{offset}}^{\prime }$) such that Eq. (5) is minimized

$$\sqrt{\underset{\lambda }{\sum }(R_{t}(\lambda )-R_{r}(\lambda ){)}^{2}}.$$ (5)

The normalized-root-mean square error (NRMSE) function can be used to determine the unmixing accuracy while normalizing using the mean ${\mathrm{HbO}}_{2}$ and Hb values $\overset{¯}{c_{{\mathrm{HbO}}_{2}}}$ and $\overset{¯}{c_{\mathrm{Hb}}}$, respectively:

$$\mathrm{NRMSE}=\frac{1}{p\cdot \overset{¯}{c_{\mathrm{Hb}}}\cdot \overset{¯}{c_{{\mathrm{HbO}}_{2}}}}\sqrt{\underset{p}{\sum }{\left(c_{\mathrm{Hb}}^{*}-c_{\mathrm{Hb}}^{**}\right)}^{2}+{\left(c_{{\mathrm{HbO}}_{2}}^{*}-c_{{\mathrm{HbO}}_{2}}^{**}\right)}^{2}},$$ (6)

where p is the number of pixels; in the spectral optimization, it is the full dataset, while in the spatial optimization it is the demosaicked hypercube. $c_{{\mathrm{HbO}}_{2}}^{*}$ and $c_{\mathrm{Hb}}^{*}$ are the concentrations of ${\mathrm{HbO}}_{2}$ and Hb calculated using the full spectra, whereas $c_{{\mathrm{HbO}}_{2}}^{**}$ and $c_{\mathrm{Hb}}^{**}$ are the concentrations of ${\mathrm{HbO}}_{2}$ and Hb calculated using the reduced spectra of interest at the selected center wavelengths and bandwidths.

The oxygen saturation (${\mathrm{sO}}_{2}$) of the different tissue types was calculated as a ratio of the concentration of ${\mathrm{HbO}}_{2}$ to the sum of ${\mathrm{HbO}}_{2}$ and Hb:

$${\mathrm{sO}}_{2}=\frac{c_{{\mathrm{HbO}}_{2}}}{c_{\mathrm{Hb}}+c_{{\mathrm{HbO}}_{2}}},$$ (7)

$$v_{\text{blood}}=c_{\mathrm{Hb}}+c_{{\mathrm{HbO}}_{2}}.$$ (8)

The relative fraction of blood $v_{\text{blood}}$ was also calculated and plotted against the ${\mathrm{sO}}_{2}$ for the different tissue types.

### 2.8 Classification Accuracy When Using the Full Spectra

To assess the compromise in classification accuracy when using a limited number of bands for MSI, we compared to classification of the tissue using hyperspectral imaging. A hyperspectral filter with center wavelengths from 470 and 720 nm in 1 nm steps was applied to the spectra with bandwidths (FWHM) of 1 to 30 nm in 1 nm steps (Fig. S2 in the Supplementary Material). When machine learning was used for classification for the kNN and SVM techniques, it was done in a fivefold method, in line with the classification techniques used on the hypercubes. The resulting classification accuracy for each tissue type and classification method was calculated following spectral band optimization in the Opti-MSFA toolbox; spatial optimization was not relevant in this case.

## 3 Results

### 3.1 Optimized Filters Designed for Classification of Esophagus and Colon

The optimal 3 to 9 band MSFA arrangements for esophageal and colon datasets were determined according to classification accuracies, where high values reflect high performance when using kNN, SVM, and SAM, while low values of NMRSE for unmixing ${\mathrm{HbO}}_{2}$ and Hb are preferred. A direct comparison across both datasets for all merit functions showing optimized filter arrays and output images is available in Tables S1–S3 in the Supplementary Material. For reference, we calculated the classification accuracies also for a 250-band sample, considering the performance that could be achieved with a full hyperspectral imaging system (Table 1 and Fig. S2 in the Supplementary Material), showing overall a greater separability of the spectra in the colon dataset than the esophageal dataset.

**Table 1:** Full hyperspectral classification accuracy of esophageal and colon tissue depending on classification type.

Classification model	Maximum classification accuracy	
	Esophagus	Colon
kNN	0.848	0.999
SVM	0.811	0.997
SAM	0.245	0.995

**Fig. 3:**
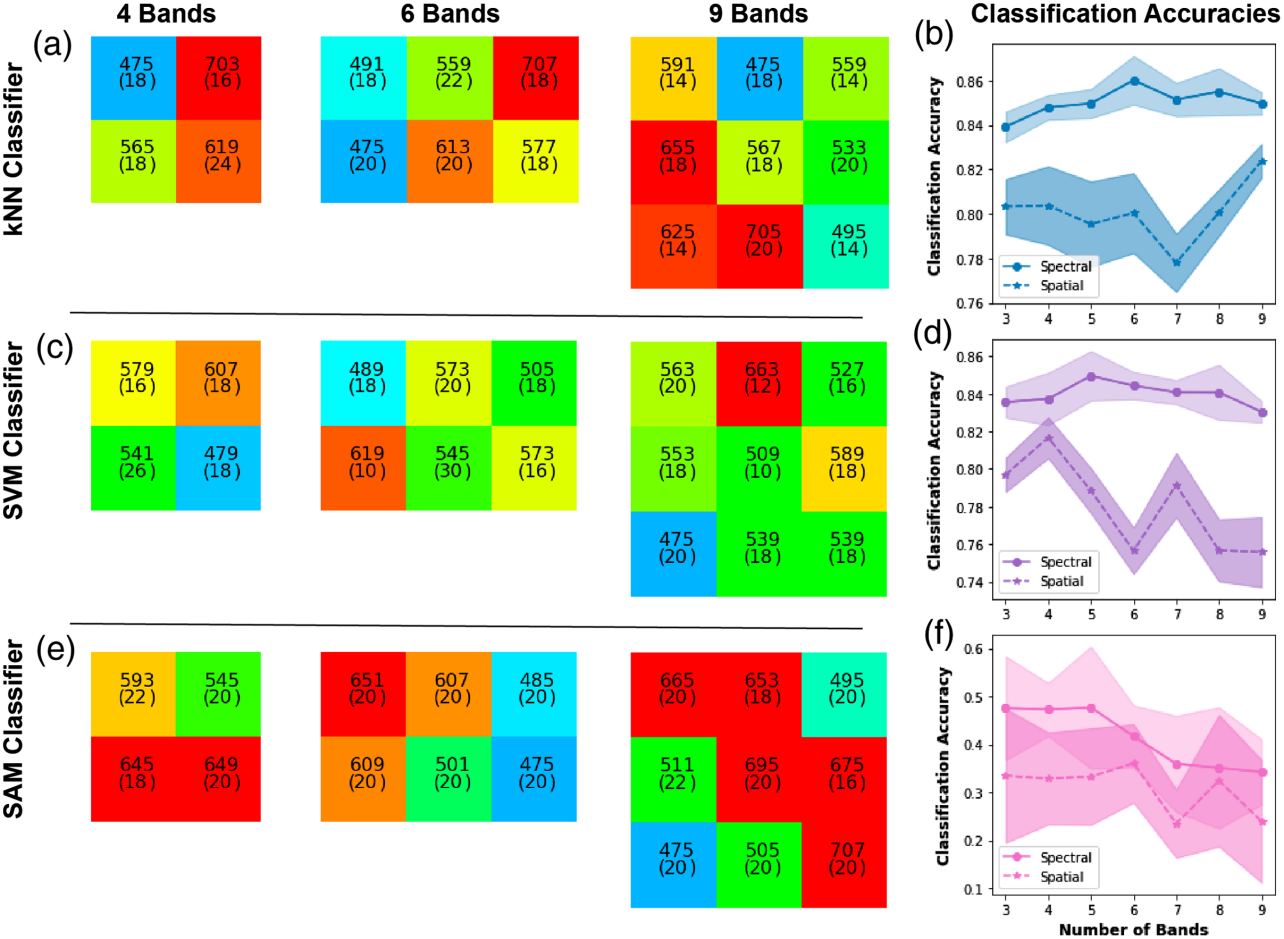
Optimal band arrangements and classification accuracy for the esophageal tissue types. Using (a), (b) kNN classification; (c), (d) SVM classification, and (e), (f) SAM classification. (a), (c), (e) The optimized spatial layouts of the filters are shown, with each colored box indicating the spectral properties, including the center wavelength and FWHM bandwidth (given in parenthesis). The spectral properties are shown in nanometers. The colors of the individual filters approximate what the human eye would see at these wavelengths. (b), (d), (f) Classification accuracies after spectral and spatial optimization and the associated 95% confidence intervals.

When considering the esophageal dataset (Tables 2 and 3), the spectral bands highlighted in the 3-band case using the kNN (475±24, 573±22, and 617±16  nm) are then generally represented in all filter sets with higher numbers of bands (Fig. 3). When adding a fourth band, a far-red region (703±16  nm) is added and again remains present through 6 and 9 band examples. The optimized outputs show consistent sampling of the hemoglobin absorption region in the 525 to 575 nm range, usually with additional bands flanking outside of this region, one towards the blue and one towards the red. A strong representation of filters in the green is also seen in the SVM results; however, in this case, the red and far-red spectral bands are only represented in the 7 to 9 band cases. For both kNN and SVM [Figs. 3(a) and 3(c)], the classification accuracy after spectral optimization is largely unaffected by adding more spectral bands [Figs. 3(b) and 3(d)], suggesting the blue-green target filters in the 3 band case are already sufficiently information rich, consistent with prior use of hemoglobin targeted narrow-band imaging approaches clinically. Spatial optimization leads to degradation in performance as would be expected (Fig. S3 in the Supplementary Material), since more spectral samples lead to poorer spatial sampling. SAM appears to make the most use of the red and far-red spectral bands [Figs. 3(e) and 3(f)] but shows poor classification accuracy in the esophageal dataset, most likely because subtle overall changes in signal intensity are important for classifying the esophageal tissue spectra and these are not accounted for in the SAM dataset.

**Table 2:** Overall classification accuracy of esophageal tissue using kNN, SVM, and SAM after spectral optimization.

Classification model	Number of filters						
	3	4	5	6	7	8	9
kNN	0.839	0.848	0.850	0.860	0.851	0.855	0.850
SVM	0.836	0.837	0.850	0.844	0.841	0.841	0.830
SAM	0.476	0.473	0.477	0.417	0.359	0.351	0.343

**Table 3:** Overall classification accuracy of esophageal tissue using kNN, SVM, and SAM after both spectral and spatial optimization.

Classification model	Number of filters						
	3	4	5	6	7	8	9
kNN	0.803	0.804	0.795	0.800	0.778	0.801	0.824
SVM	0.797	0.817	0.789	0.756	0.792	0.757	0.756
SAM	0.335	0.329	0.333	0.360	0.235	0.324	0.240

**Fig. 4:**
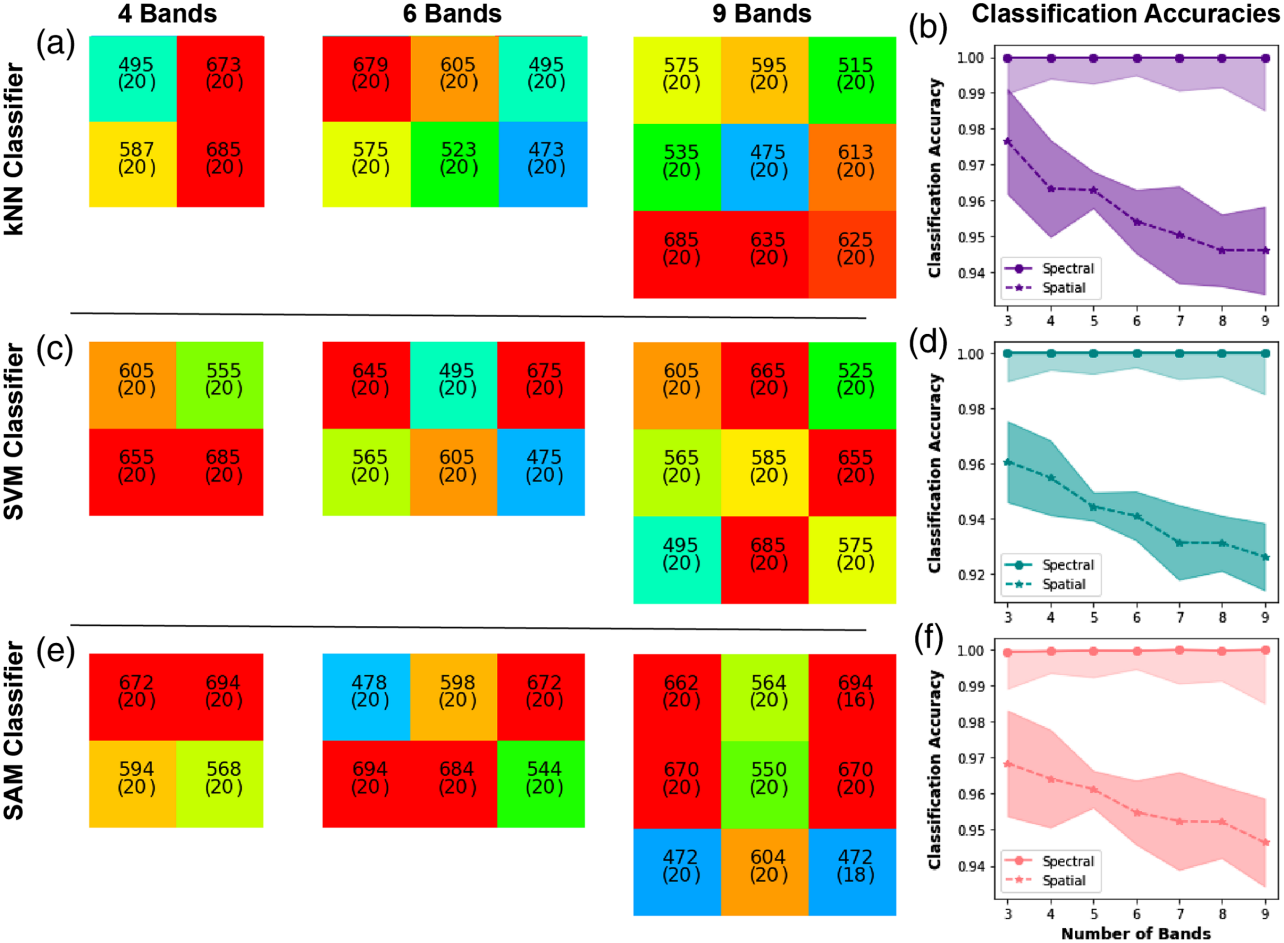
Optimal band arrangements and classification accuracy for the colon tissue types. Using (a), (b) kNN classification, (c), (d) SVM classification, and (e), (f) SAM classification. (a), (c), (e) The spatial arrangement of the filters is shown as per Fig. 4. (b), (d), (f) Classification accuracies after spectral and spatial optimization and the associated 95% confidence intervals.

A similar trend is seen for the colon dataset in terms of both the information-rich spectral features and the classification performance for kNN [Figs. 4(a) and 4(b)] and SVM [Figs. 4(c) and 4(d)]. For the colon dataset, the central region of sampling is slightly red-shifted to 550 to 600 nm range, with further sampling in the red common, particularly for SAM [Figs. 4(e) and 4(f)]. All methods show perfect or near-perfect classification accuracy (Tables 4 and 5) following the spectral optimization, owing to the more separable spectral features in the dataset, which leads to less reliance on global intensity changes. To examine this further, a 95% confidence interval was calculated by finding the average classification accuracy or unmixing error for each patient and then performing a t-test on these averages; these are shown in plots of classification accuracy and unmixing error. Interpatient variation is also lower than in the esophageal data as there is a smaller number of biological replicates (patients). After spatial optimization, the performance degrades somewhat but remains above 95% for 3- and 4-band solutions; adding more filters does not lead to improvement in performance and in fact reduces classification accuracy, suggesting this is adding noise. The results indicate that it is not necessary to go beyond 3 or 4 spectral band MSI for the classification of these datasets and to do so may detriment the outcome; however, this finding is likely due to the limited number of patients in the dataset and we would expect a performance degradation with an expanded clinical spectral dataset with a more representative sampling of interpatient variation.

**Table 4:** Overall classification accuracy of colon tissue using kNN, SVM, and SAM after spectral optimization.

Classification model	Number of filters						
	3	4	5	6	7	8	9
kNN	1.0000	1.0000	1.0000	1.0000	1.0000	1.0000	1.0000
SVM	1.0000	1.0000	1.0000	1.0000	1.0000	1.0000	1.0000
SAM	0.9993	0.9995	0.9997	0.9996	0.9999	0.9997	0.9999

**Table 5:** Overall classification accuracy of colon tissue using kNN, SVM, and SAM, after spectral and spatial optimization.

Classification model	Number of filters						
	3	4	5	6	7	8	9
kNN	0.976	0.963	0.963	0.954	0.950	0.946	0.946
SVM	0.961	0.955	0.944	0.941	0.931	0.931	0.926
SAM	0.968	0.964	0.961	0.955	0.952	0.952	0.946

### 3.2 Optimization Based on Linear Spectral Unmixing for Hemoglobin Content and Oxygenation

**Fig. 5:**
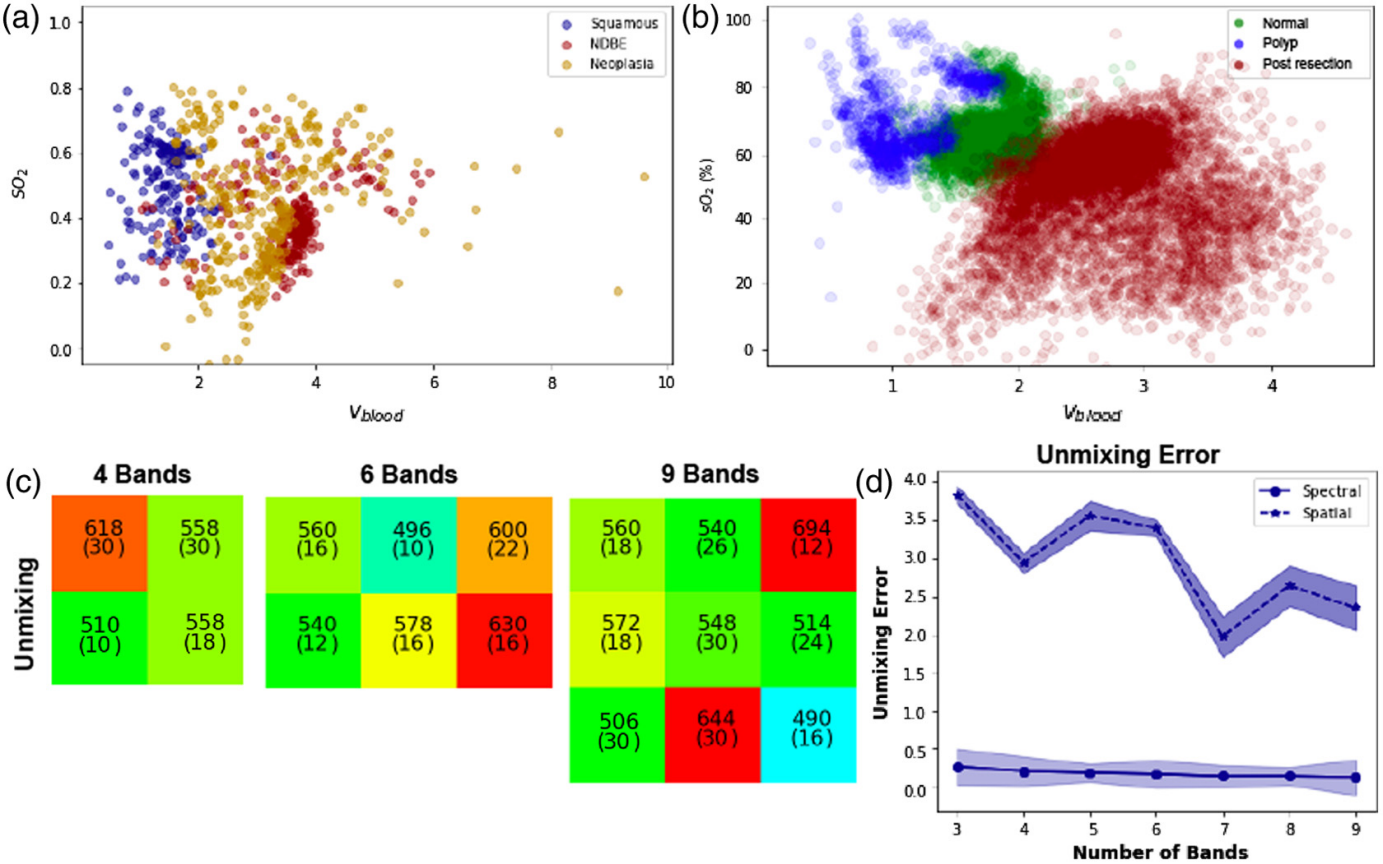
Unmixing of oxygen saturation (${\mathrm{SO}}_{2}$) and blood volume ($v_{\text{blood}}$). (a) Poor differentiation for tissue types in the esophagus is seen based on the oxygenation, consistent with prior publication. Conversely, better discrimination is seen (b) for the various tissue types in the colon, hence the colon dataset only was subjected to MSFA optimization based on linear unmixing. (c) The filters optimized using unmixing on colon tissue types. (d) Unmixing error after spectral and after spatial optimization and the associated 95% confidence intervals.

We next examined the potential to apply linear spectral unmixing to the recorded spectra for the assessment of physiological parameters of hemoglobin content and oxygenation and using the NRMSE to determine the goodness of fit with different numbers of filters. Consistent with prior findings,^8^ the esophageal dataset was not clearly separable based on these parameters [Fig. 5(a)]; however, the colon dataset, newly examined using this approach, was highly separable [Fig. 5(b)]. Not only was it possible to resolve postresection bleeding from polyps, it was also possible to distinguish polyps from normal tissue using these metrics. We therefore proceeded to undertake spectral band optimization only for the colon dataset.

The goodness of fit for the measured reflectance spectra to reference spectra for constituent oxy- and deoxyhemoglobin components, along with water and background scattering, was first examined across each colon tissue type. Overall, the fit performance was high for normal tissue, ranging from $r^{2}=0.82$ to 0.99 (Table S4 in the Supplementary Material), but as expected showed a greater variance in the diseased states, ranging from $r^{2}=0.54$ to 0.99 in polyp tissue (Table S5 in the Supplementary Material) and $r^{2}=0.48$ to 0.99 in postresection tissue (Table S6 in the Supplementary Material). The majority of the fits are in the upper $r^{2}$ range and the few lower performing fits are due to noise in the spectra (Figs. S4–S6 in the Supplementary Material). Undertaking the optimization process on these spectra [Fig. 5(c)] again shows the need for strong sampling in the green, but now flanked by a bluer and redder band (510±10, 558±18, and 618±30  nm). Interestingly, the red band in this case is rather broad compared to the other merit functions, perhaps linked to the fit to the background scattering term. The unmixing error declines with increasing number of bands for spectral unmixing, indicating that it is easier to fit the reference spectra with increased sampling [Fig. 5(d) and Table 6].

**Table 6:** Spectral unmixing NRMSE of colon tissue after spectral and spatial optimization.

Classification type	Number of filters						
	3	4	5	6	7	8	9
After spectral optimization	0.250	0.192	0.170	0.153	0.125	0.127	0.107
After spatial optimization	3.832	2.942	3.556	3.395	1.975	2.644	2.354

### 3.3 Filter Classification Illustrated on Synthetic Hypercubes

**Fig. 6:**
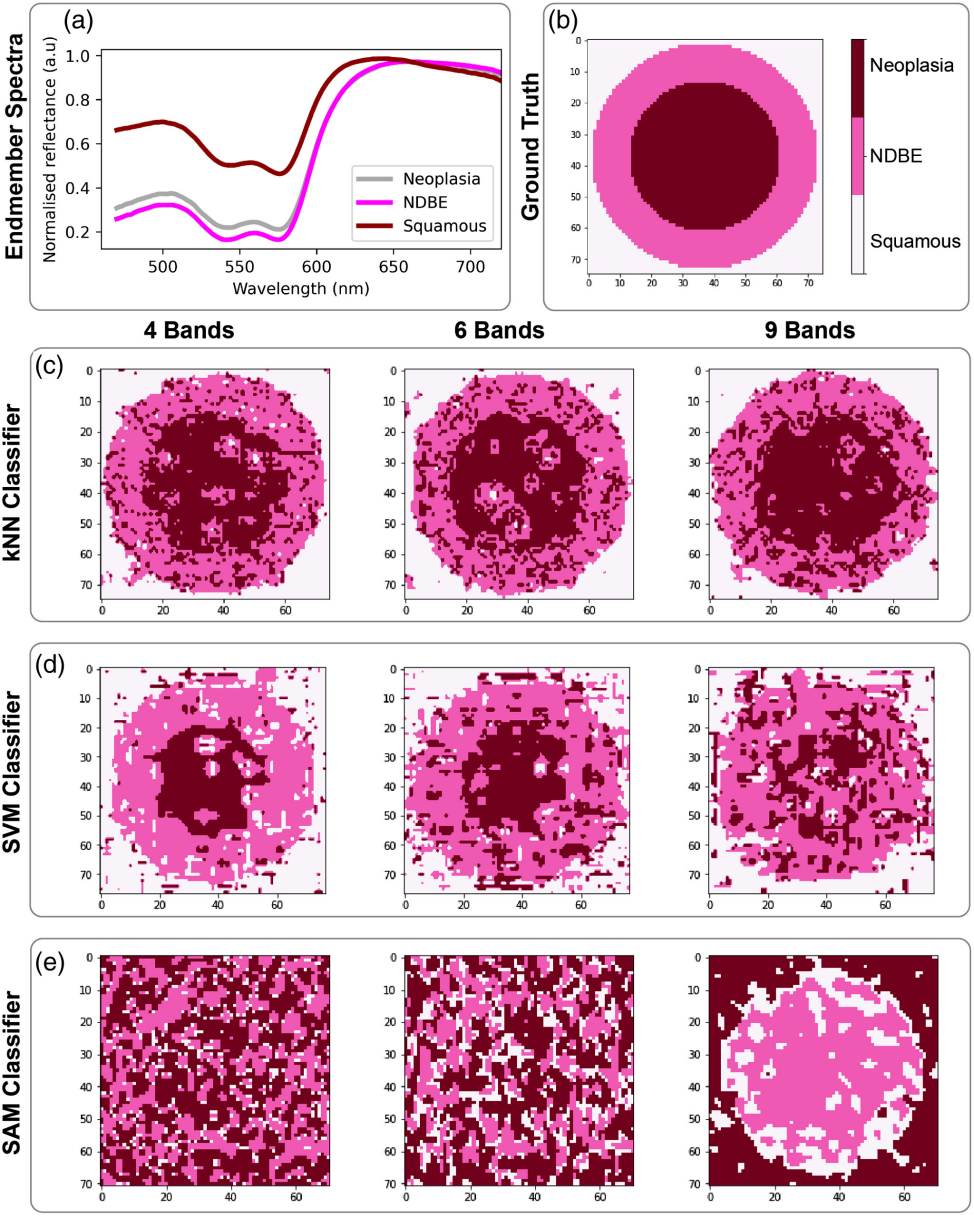
Esophageal classification imaging outputs. (a) The spectral endmembers and (b) the ground truth of the synthetic hypercubes used in the optimization process. The resulting synthetic hypercube image classifications using the different MSFA designs are shown for (c) kNN, (d) SVM, and (e) SAM.

Overall, the output results from Opti-MSFA indicate that both kNN and SVM classification had similar accuracies in esophageal tissue, reflecting the higher interpatient variation in the esophageal dataset.^8^ The classification accuracy of the colon tissue was much higher as the colon spectra are more separable, as found in prior examination using SAM.^9^ We next explored how the optimal choice of spectral bands would translate into imaging performance. In the esophageal dataset (Fig. 6), the reconstruction of the concentric circles of different tissue types is well reflected in the kNN and SVM, with some “spillover” at the boundaries between regions. A higher level of misclassification of the normal tissue is seen using SVM. The SAM approach gives a poor performance, with a circular structure only barely apparent in the 9-band case and even then showing an inversion of the disease types spatially. The performance of the MSFA-based approach clearly decreases as spatial resolution is lost in the 6-band and 9-band mosaic patterns.

**Fig. 7:**
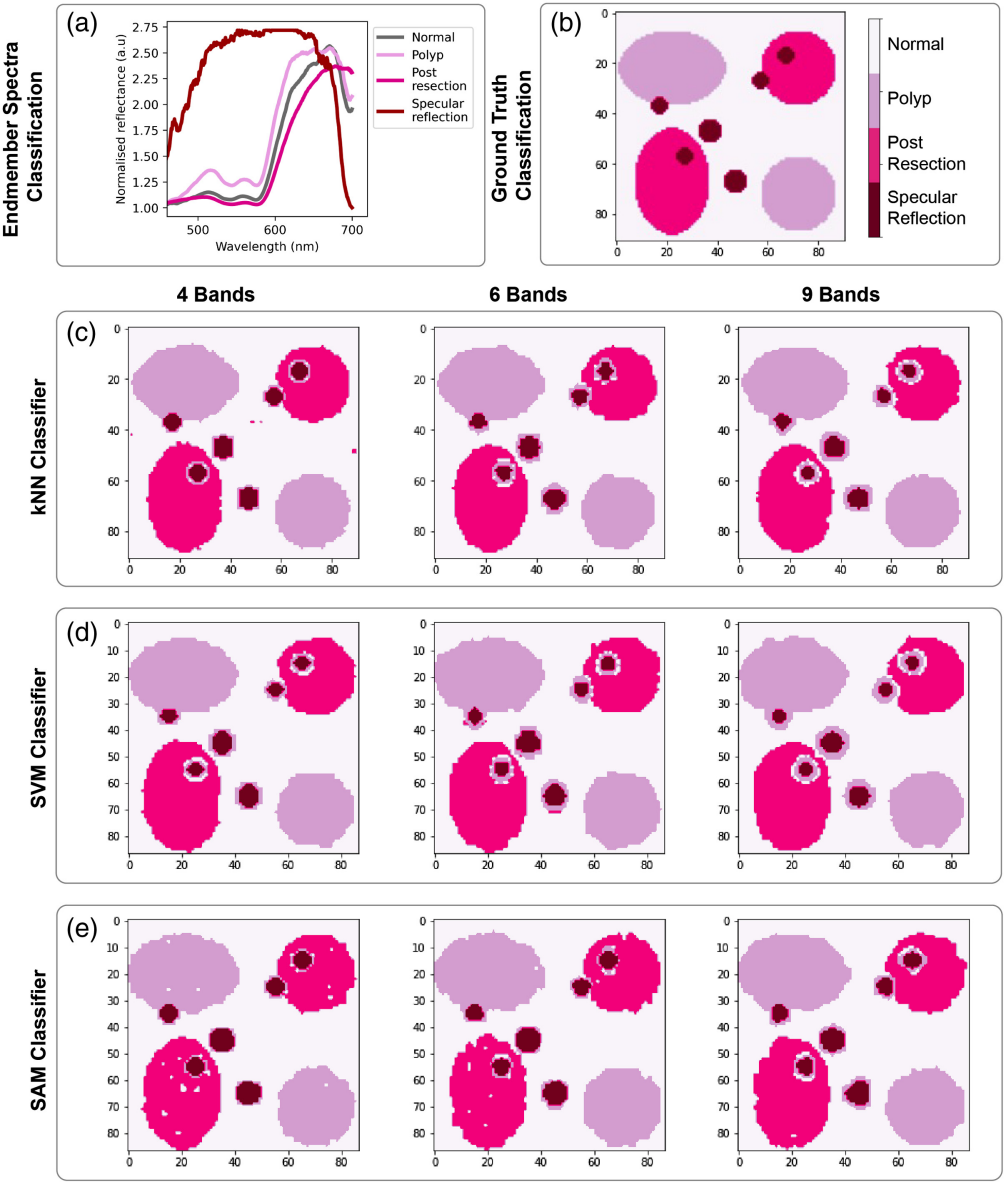
Colon classification imaging outputs. (a) The spectral endmembers and (b) the ground truth of the synthetic hypercubes used in the optimization process. The resulting synthetic hypercube image classifications using the different MSFA designs are shown for (c) kNN, (d) SVM, and (e) SAM.

**Fig. 8:**
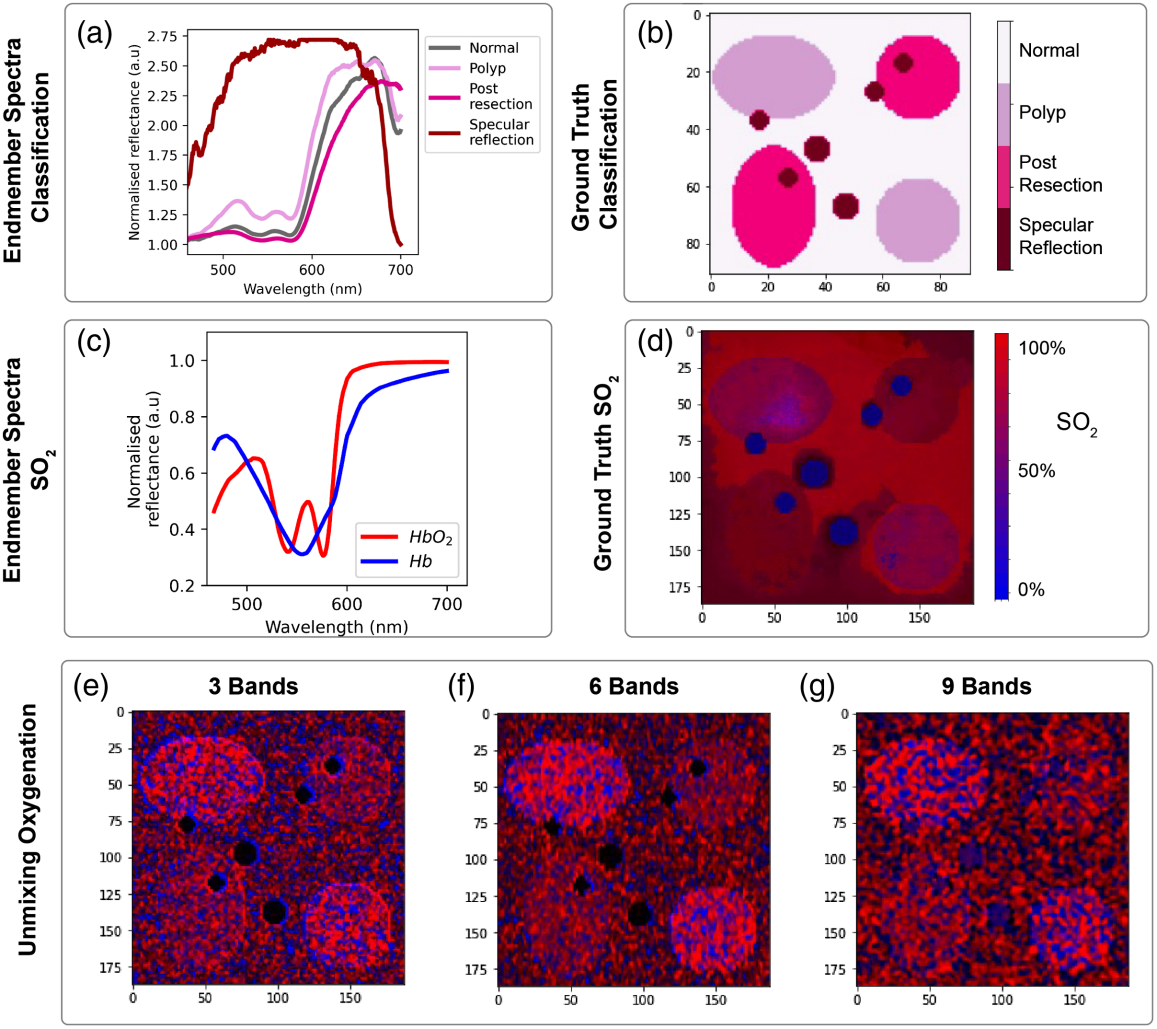
Colon spectral unmixing imaging outputs. (a) The spectral endmembers and (b) ground truth of the synthetic hypercubes used in the classification optimization process are illustrated. (c) The corresponding spectral endmembers and (d) ground truth in the unmixing of ${\mathrm{SO}}_{2}$ are illustrated. (e)–(g) The unmixing ${\mathrm{SO}}_{2}$ abundance maps for the MSFA optimization are also shown.

In the colon dataset (Fig. 7), classification performance is again degraded at the edges of features, with poorer edge definition seen with an increasing number of filters. Interestingly, the edges of the specular reflections are consistently misclassified first to polyp and then to normal tissue with distance from the center. This would be important clinically since specular reflections are image artifacts and could be misleading if classified as polyp tissue. In the case of unmixing (Fig. 8), spectral resolution is more important and outweighs the deterioration due to spatial resolution (Table S7 in the Supplementary Material).

## 4 Discussion

Here we applied an open-source software toolbox Opti-MSFA to analyze published datasets with the goal of identifying the potential for MSI to enhance contrast during endoscopy for early cancer detection. Our analysis of the optimal wavelengths and bandwidths for discrimination of early cancerous lesions in the GI tract shows promise, even with only 3 or 4 targeted wavebands, although it should be noted that the interpatient variation within the datasets under test was relatively limited. The classification accuracies achieved for a highly restricted bandset were very similar to those obtained for the full hyperspectral dataset, indicating that MSI using information-rich spectral bands presents a good strategy to trading spectral and spatial information.

Testing the MSI approach across different merit functions and datasets revealed some interesting insights. In both datasets, there was typically a central spectral sampling range in which one or more spectral band appeared, flanked by a bluer and a redder spectral band. Bands present in the 3-band case then appeared similarly as an optimization output as the number of bands increased. The precise location of these bands varied slightly between the esophageal and colon datasets, with the central sampling range being 525 to 575 nm in the former and 550 to 600 nm in the latter.

The bandwidth of the targeted spectral bands was generally quite broad in all cases, on the order of 20 nm, likely due to the presence of relatively smoothly varying spectral features. It is likely that the wider bandwidth balances the trade-off of improved spectral resolution that occurs with a small bandwidth and the additional noise that occurs due to the reduced amount of light that reaches the image sensor, since everything outside of this band is rejected. This was not the case when the unmixing accuracy of ${\mathrm{HbO}}_{2}$ and Hb was calculated, with spectral bandwidths as small as 10 nm and as large as 30 nm, the minimum and maximum bandwidths allowed, indicating that spectral sensitivity is important when unmixing ${\mathrm{HbO}}_{2}$ and Hb. The use of wider bands is advantageous from the perspective of optical system design for future applications, requiring less stringent design specifications for optical components, but may not be tolerated for applications where unmixing for physiological metrics is preferred over disease classification.

Finally, the trade-off between addition of spectral information and reduction in spatial resolution must be considered. The classification accuracy after spatial optimization for both datasets generally decreased as the number of bands in the filters increased, except for spectral unmixing where the NRMSE improved with more bands. It is worth noting that spectral optimization assumes a complete hypercube can be measured, whereas the spatial optimization results in a sparse hypercube due to the MSFA. With the classification models, as the number of bands increases, there is a higher chance of adding a less information rich, noisier, band and the lost spatial information then outweighs the additional spectral information. Some variation is seen according to the optimization metric used. Although SAM is the simplest and least computationally intensive method, it relies on distinct spectral-rather than intensity-based changes, which means that it shows a poor performance in the esophageal dataset, where the changes between tissue types are more heavily reliant on intensity changes. Comparing kNN and SVM, kNN can better discern the edges of the neoplastic or polyp regions against the background. The decrease in classification accuracy with number of bands is seen most significantly in the SVM classifiers, where increasing the number of bands from 3 to 9 reduces accuracy by 0.08, while the kNN classifier only decreases by 0.007. These findings reinforce the importance of choosing an appropriate classifier for the dataset under investigation, considering spatial and spectral information, and noise characteristics. Further work is necessary to fully understand the best choice of merit function for a given set of data and how it will influence the performance of the resulting MSFA. SAM performed well on the colon dataset, comparable to kNN and SVM classification methods, but very poorly on the esophageal dataset. This is likely because SAM has a limited discriminative power and struggles with the tissue types are similar or spectra that are complex mixtures. By comparison, kNN and SVM are better able to handle complex and nonlinear decision boundaries that SAM is unable to and are generally more robust to noise.

Although the analysis performed here is promising for the use of targeted spectral bands in the GI tract, it is based on model hypercubes using a relatively restricted set of published spectra. The relatively high performance for a small number of spectral bands indicates that there are distinct spectral regions (particularly linked to hemoglobin absorption) that explain most of the variance between the disease states; adding further spectral sampling beyond these regions also adds noise, which can detriment classification. Further analysis should evaluate how inter- and intrapatient variation, along with noise variations in imaging systems, might affect the optimal filter arrays in a larger dataset. The testing and training datasets here were split before training, but since there is interpatient variation, this could lead to overclassification. To assess this, the 95% confidence interval was calculated on the average classification accuracies of different patients. The highest classification accuracies occurred in the colon dataset, in agreement with the classification in previous work that used SAM methods and showed very good separation of the spectra of the different tissue types. Further work could assess how these classification accuracies vary between patients and consider confounding factors that may influence the measurements, such as age or genetics. To assess the impact of the spatial variation in true hyperspectral data, future work should acquire full hyperspectral imaging datacubes from endoscopy *in situ* to optimize MSFAs, rather than using spectroscopy data to simulate the imaging scenario. Other sources of error that would be encountered in a clinical setting, such as patient motion, should also be examined. Nevertheless, the results shown here demonstrate that the complex trade-offs involved with spectral imaging can be balanced by tuning the MSFAs using different merit functions to focus on classification or unmixing.

Furthermore, the study highlights the necessity of addressing interpatient variability and the potential for overclassification when a dataset arising from a limited number of biological replicates (i.e., patients) is used. The promising classification accuracies observed underscore the effectiveness of the spectral band selection, but it is important to ensure that these results are not solely due to the constraints of the dataset. Ongoing work to acquire data from a larger number of patients to fairly reflect biological variations and additionally acquire *in situ* hyperspectral imaging data cubes will allow deeper analysis into the complex trade-offs that spectral imaging presents. Ultimately, the ability to fine-tune the MSFAs with methods that are robust and clinically applicable amidst the diverse variables presented by actual patient data and the clinical environment has the potential to improve patient outcomes by enabling early cancer detection.

## 5 Conclusion

Customized MSFAs with a relatively small number of spectral bands could be applied to enhance contrast for early cancer detection in the GI tract. Using targeted spectral bands, classification of different tissues can be optimized to improve early cancer diagnosis. Future work includes testing filters in a clinical setting and collecting more spectral datasets from a larger number and diversity of patients to improve the design of endoscopes for use in the GI tract.

## Supplementary Material



## Data Availability

The code and synthetic datacubes used in the generation of this article are available at: https://doi.org/10.17863/CAM.99953.
