# Supplementary material for: Targeted multispectral filter array design for the optimization of endoscopic cancer detection in the gastrointestinal tract
Source: J Biomed Opt. 2024 Mar 29;29(3):036005. doi: 10.1117/1.JBO.29.3.036005 (PMC10978444; doi:10.1117/1.JBO.29.3.036005)
Supplement: Supplementary file 1 [file JBO_029_036005_SD001.pdf]

Supplementary Information for: Targeted Multispectral Filter Array Design for Endoscopic Cancer Detection in the Gastrointestinal Tract

Michaela Taylor-Williams[1,2], Ran Tao[1,2], Travis W Sawyer[3], Dale Waterhouse[4], Jonghee Yoon[5], Sarah E Bohndiek[1,2]*

1 Department of Physics, Cavendish Laboratory, University of Cambridge, JJ Thomson Avenue, Cambridge, CB3 0HE, UK
2 Cancer Research UK Cambridge Institute, University of Cambridge, Robinson Way, Cambridge, CB2 0RE, UK
3 Wyant College of Optical Sciences, University of Arizona, Tucson, USA
4 Wellcome/EPRSC Centre for Interventional and Surgical Sciences (WEISS), University College London, Gower Street, London, WC1E 6BT, UK
5 Department of Physics, Ajou University, 16499, South Korea

* Correspondence: Sarah E Bohndiek, Department of Physics, Cavendish Laboratory, University of Cambridge, JJ Thomson Avenue, Cambridge, CB3 0HE, UK and Cancer Research UK Cambridge Institute, University of Cambridge, Robinson Way, Cambridge, CB2 0RE, UK; seb53@cam.ac.uk; phone +441223 337267.

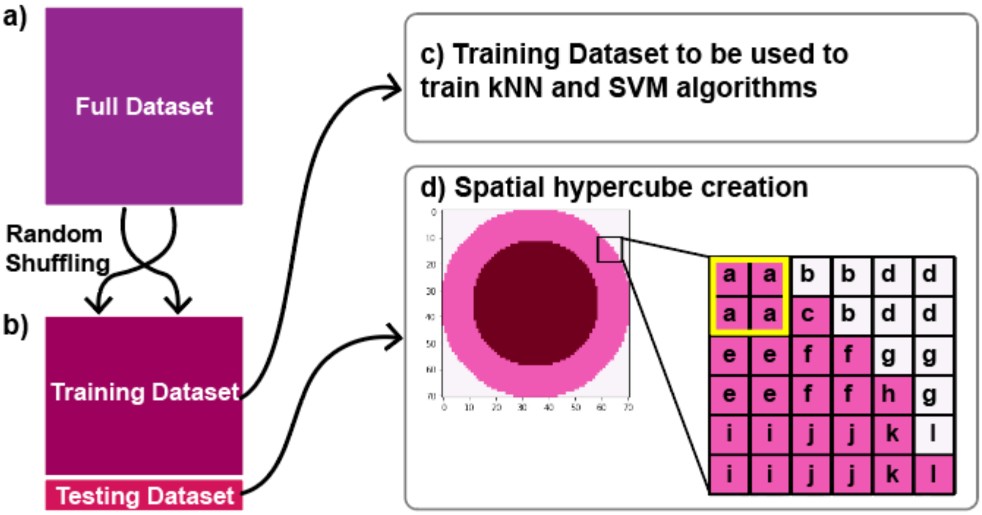

Supplementary Figure 1. The process of synthetic hypercube creation. (a) Hypercubes use with the full data set or a randomly shuffled data subset split in testing and training datasets (for kNN and SVM classification). **(b)** The training data set is used to train the algorithm of interest and **(c)** the testing dataset is used to make a spatial hypercube, such that 2x2 regions have the same spectra as shown by the letters illustrating different spectral data randomly selected from the testing data set.

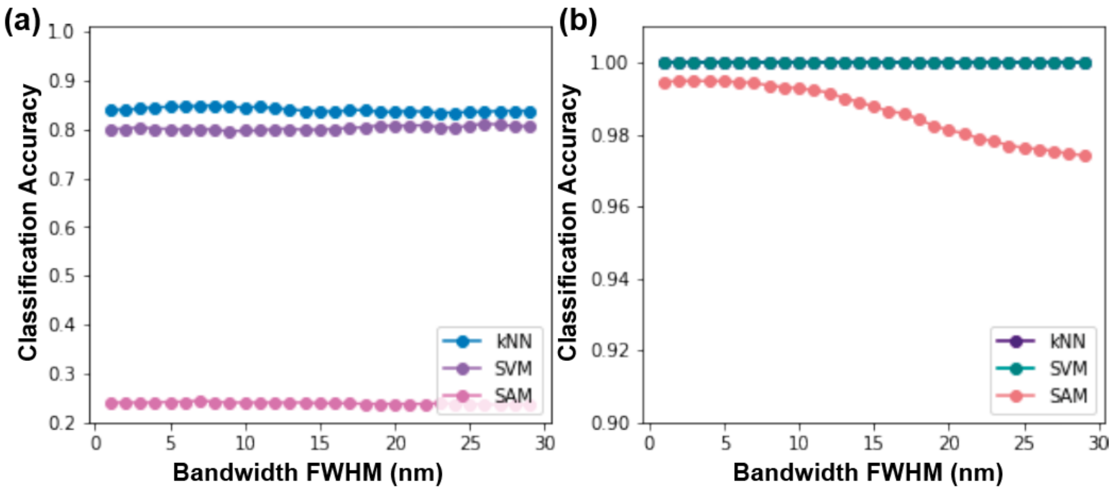

Supplementary Figure 2. Classification of (a) oesophageal and (b) colon tissue with 250 filters with varying bandwidths. Classification accuracies were calculated for 250 bands (from 470 to 720 nm). The bandwidths of the filters were then assessed from 1 to 30 nm. The classification accuracy of the colon using kNN equivalent to the SVM, so the data points are not visible.

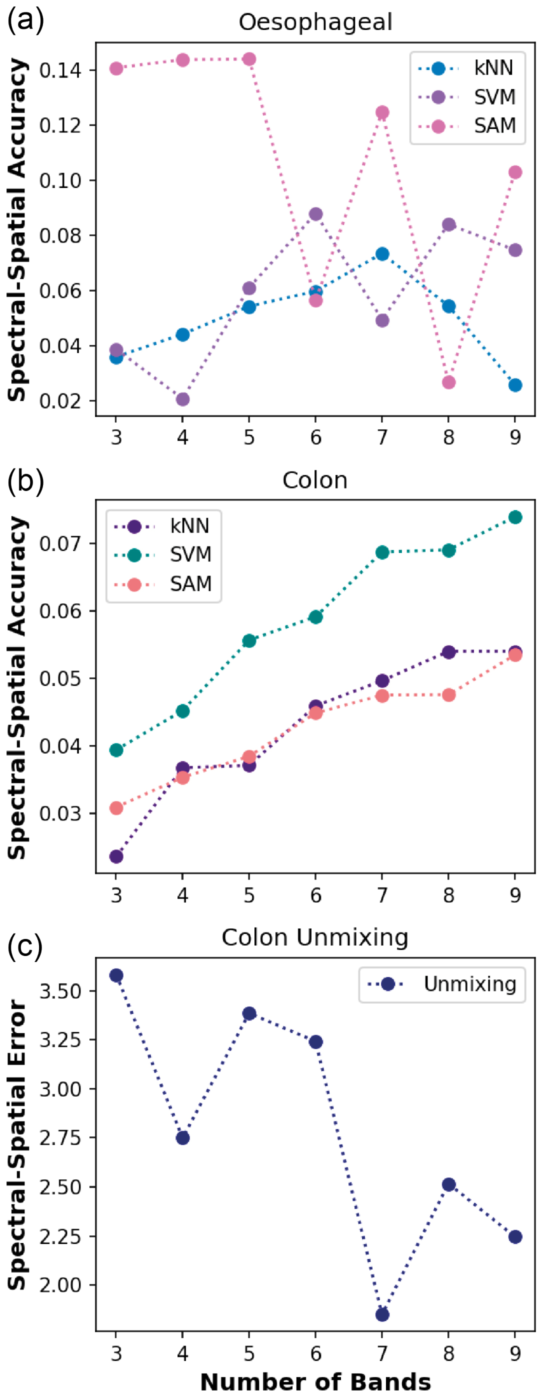

Supplementary Figure 3. The difference between the spatial and spectral classification accuracies for **(a)** oesophageal and **(b)** colon tissue is shown. The equivalent for unmixing of the colon is shown **(c)** but the difference between the spectral and spatial classification accuracies is shown since a lower unmixing error is desired. Note that a higher classification accuracy is desired **(a,b)** but in the case of unmixing error **(c)** a lower value indicates better performance and less error.

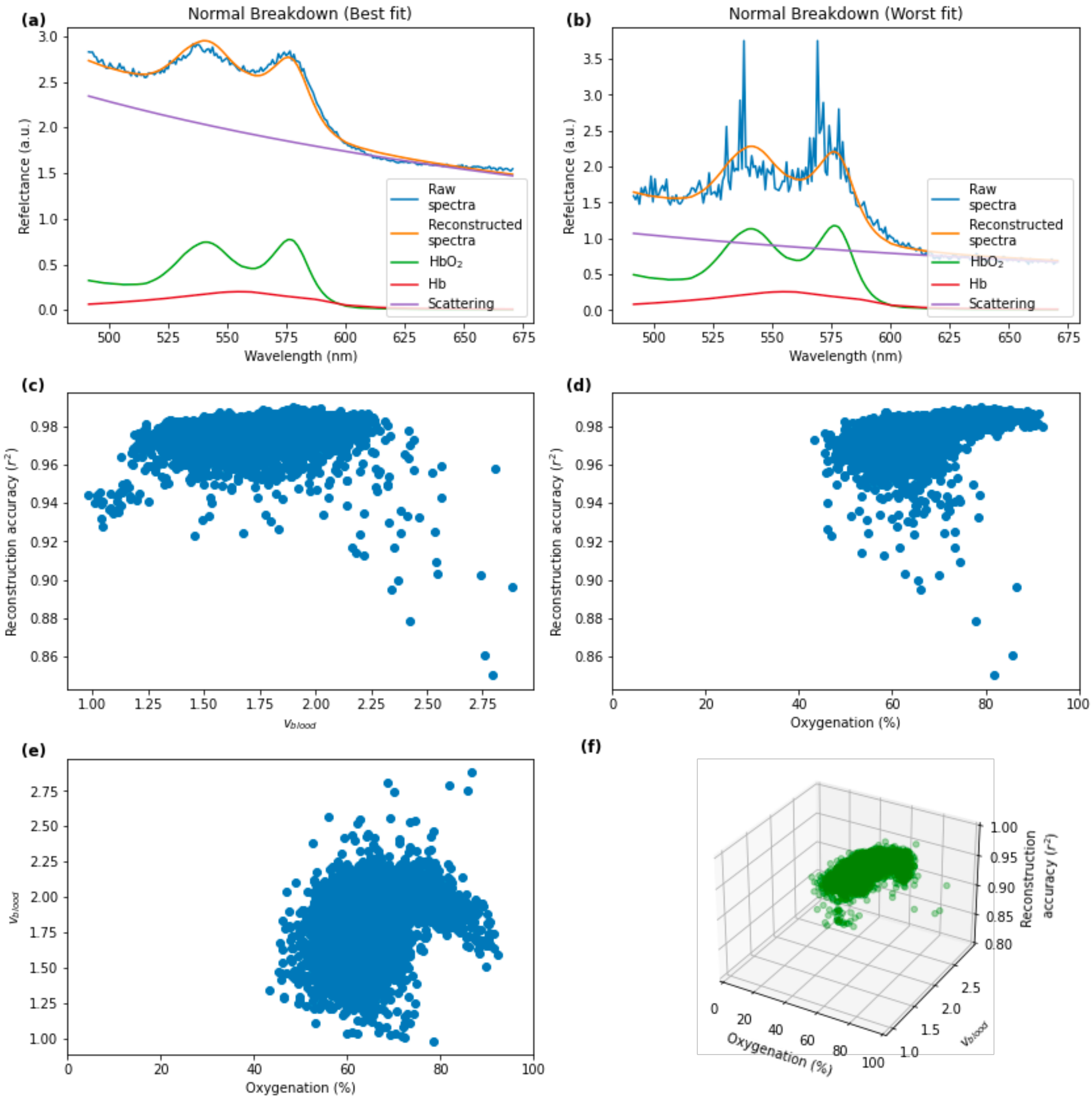

Supplementary Figure 4: Normal tissue spectral fitting (a) Raw and reconstructed spectra for the sample for the best fit example. The associated HbO_2, Hb, and scattering components that make up the reconstruction are shown. Please note the offset has been removed from both raw and reconstructed data to enable appropriate comparison; this was done for all further plots of raw and reconstructed spectra. (b) Raw and reconstructed spectra for the sample with the worst fit. (c) Reconstruction accuracy compared to the amount of blood (sum of Hb and HbO_2 values) for all normal tissue. (d) Reconstruction accuracy compared for the calculated oxygenation. (e) The amount of blood and oxygenation are compared. (f) A 3D plot illustrating reconstruction accuracy, the amount of blood, and oxygenation of normal tissue spectra.

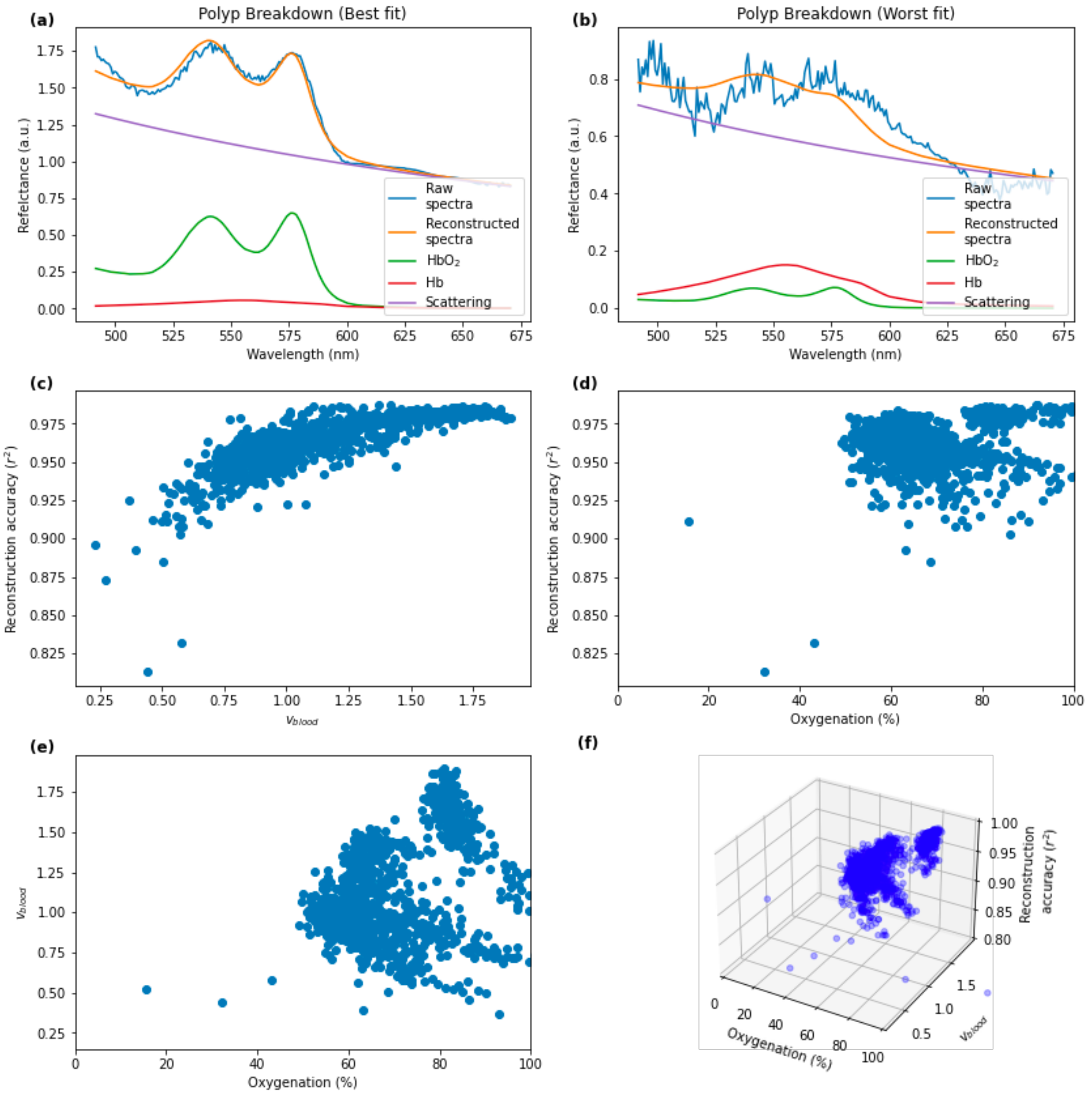

Supplementary Figure 5: Polyp tissue spectral fitting (a) Raw and reconstructed spectra for the sample for the best fit example. The associated HbO_2, Hb, and scattering components that make up the reconstruction are shown. (b) Raw and reconstructed spectra for the sample with the worst fit. (c) Reconstruction accuracy compared to the amount of blood (sum of Hb and HbO_2 values) for all normal tissue. (d) Reconstruction accuracy compared for the calculated oxygenation. (e) The amount of blood and oxygenation are compared. (f) A 3D plot illustrating reconstruction accuracy, the amount of blood, and oxygenation of normal tissue spectra.

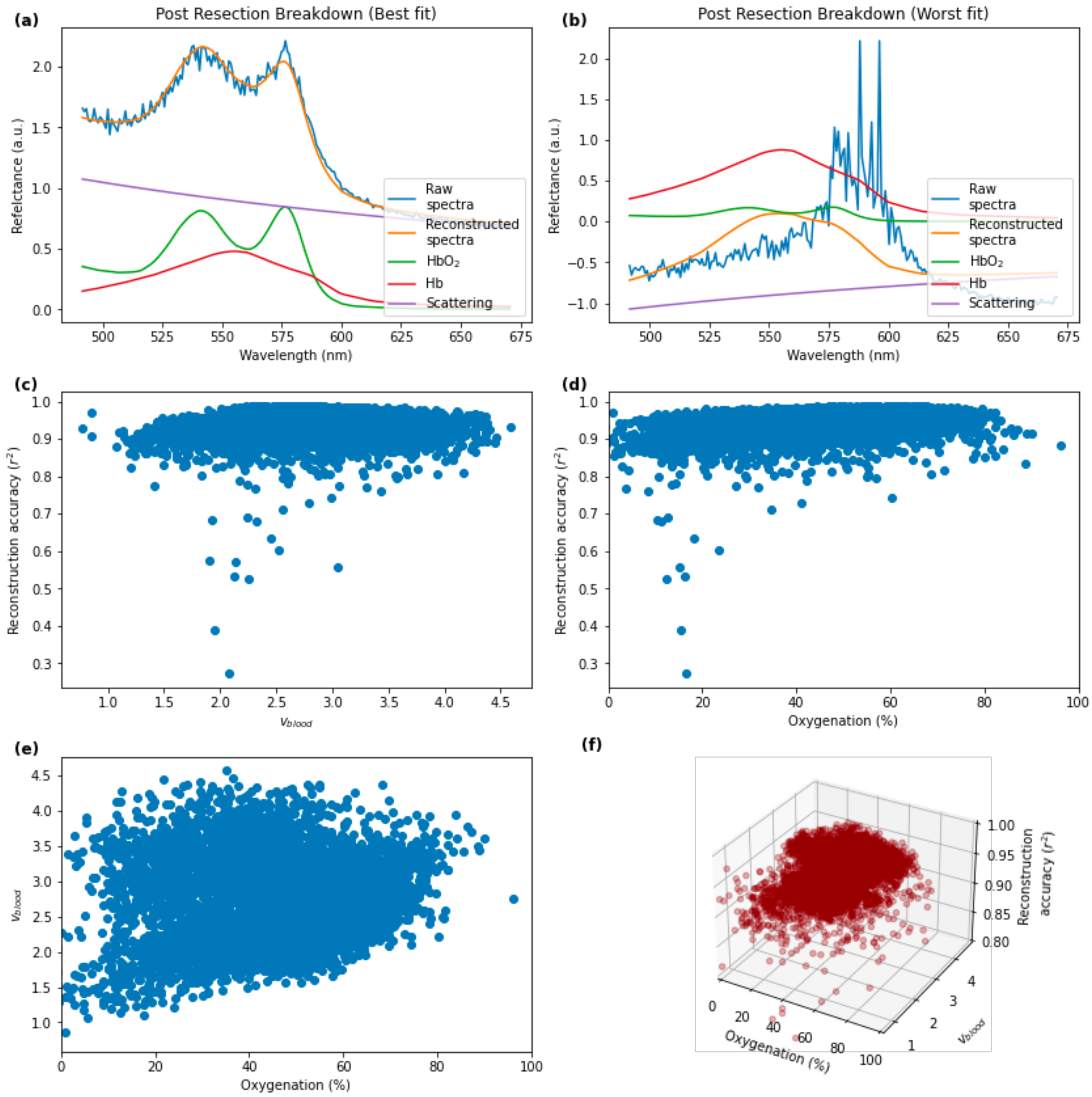

Supplementary Figure 6: Post-resection tissue spectral fitting. (a) Raw and reconstructed spectra for the sample for the best fit example. The associated HbO_2, Hb, and scattering components that make up the reconstruction are shown. (b) Raw and reconstructed spectra for the sample with the worst fit. (c) Reconstruction accuracy compared to the amount of blood (sum of Hb and HbO_2 values) for all normal tissue. (d) Reconstruction accuracy compared for the calculated oxygenation. (e) The amount of blood and oxygenation are compared. (f) A 3D plot illustrating reconstruction accuracy, the amount of blood, and oxygenation of normal tissue spectra.

Supplementary Table 1: **kNN Classification of oesophageal and colon data set.** The optimised MSFA for the oesophageal dataset, and the resulting classification of the synthetic hypercube are shown in the second and third column, respectively. The optimised MSFA for the colon dataset, and the resulting classification of the synthetic hypercube are shown in the fourth and fifth column, respectively.

No of Bands	Oesophageal MSFA	Oesophageal Classification	Colon MSFA	Colon Classification
**3**	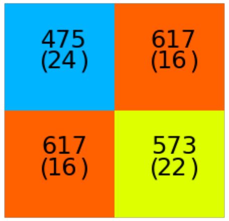	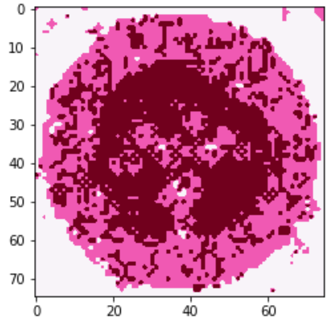	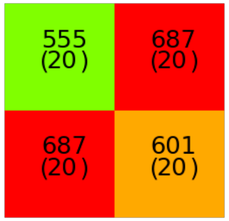	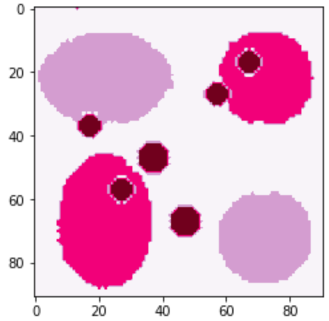
**4**	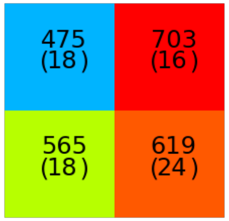	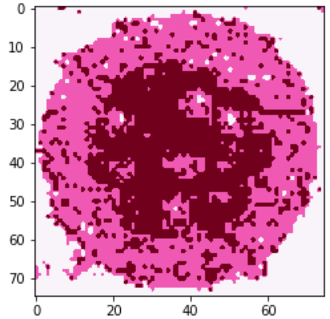	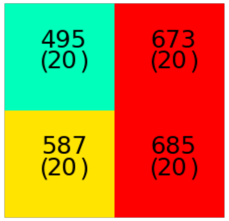	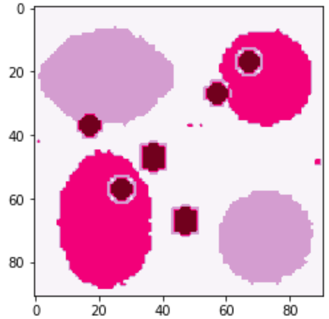

**5**	569 (18) · 701 (22) · 617 (24)<br>475 (20) · 617 (24) · 547 (10)	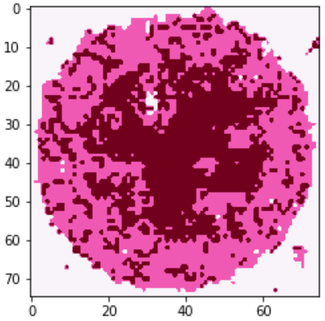	595 (20) · 475 (20) · 475 (20)<br>555 (20) · 605 (20) · 685 (20)	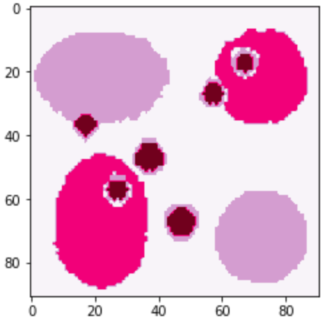
**6**	491 (18) · 559 (22) · 707 (18)<br>475 (20) · 613 (20) · 577 (18)	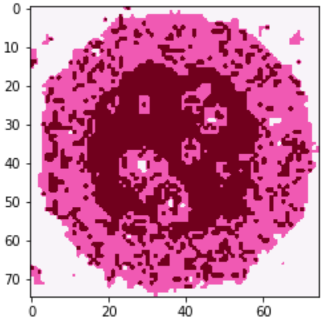	679 (20) · 605 (20) · 495 (20)<br>575 (20) · 523 (20) · 473 (20)	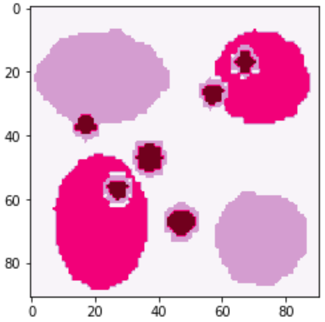
**7**	549 (20) · 703 (20) · 661 (14)<br>615 (18) · 615 (18) · 615 (18)<br>475 (12) · 489 (22) · 565 (22)	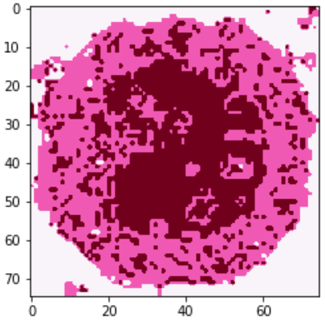	613 (20) · 555 (20) · 455 (20)<br>585 (20) · 675 (20) · 455 (20)<br>455 (20) · 667 (20) · 483 (20)	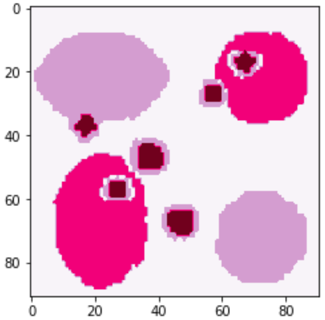

**8**	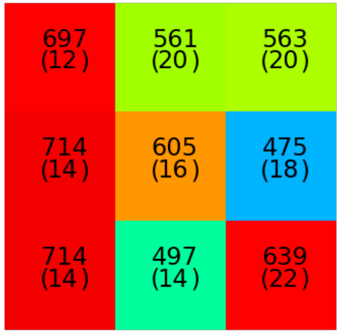697 (12)<br>561 (20)<br>563 (20)<br>714 (14)<br>605 (16)<br>475 (18)<br>714 (14)<br>497 (14)<br>639 (22)	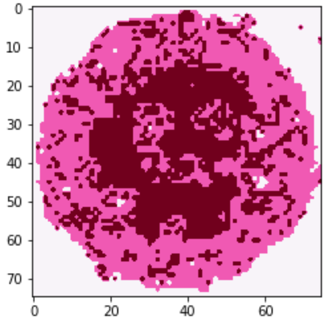0<br>10<br>20<br>30<br>40<br>50<br>60<br>70<br>0<br>20<br>40<br>60	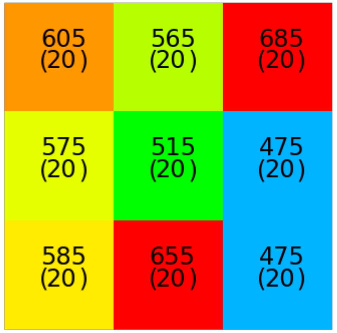605 (20)<br>565 (20)<br>685 (20)<br>575 (20)<br>515 (20)<br>475 (20)<br>585 (20)<br>655 (20)<br>475 (20)	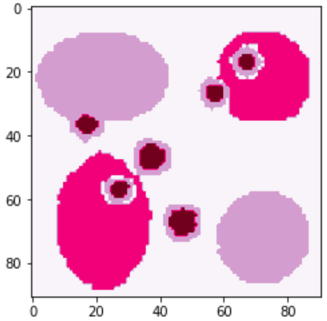0<br>20<br>40<br>60<br>80<br>0<br>20<br>40<br>60<br>80
**9**	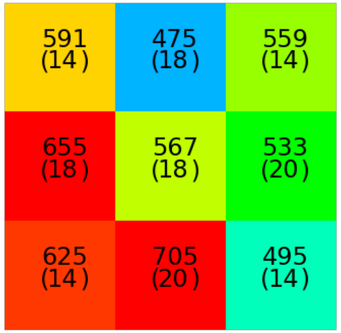591 (14)<br>475 (18)<br>559 (14)<br>655 (18)<br>567 (18)<br>533 (20)<br>625 (14)<br>705 (20)<br>495 (14)	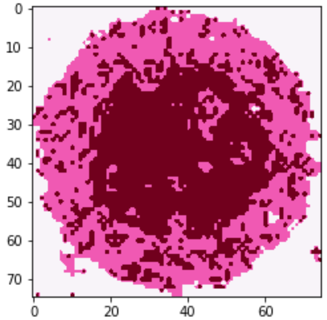0<br>10<br>20<br>30<br>40<br>50<br>60<br>70<br>0<br>20<br>40<br>60	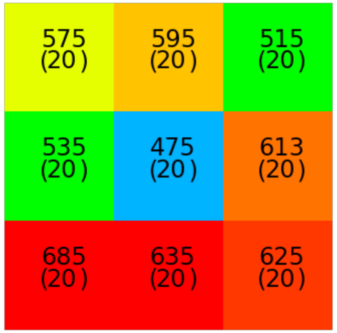575 (20)<br>595 (20)<br>515 (20)<br>535 (20)<br>475 (20)<br>613 (20)<br>685 (20)<br>635 (20)<br>625 (20)	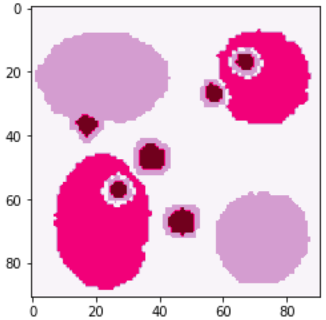0<br>20<br>40<br>60<br>80<br>0<br>20<br>40<br>60<br>80

Supplementary Table 2: **SVM Classification of oesophageal and colon data set.** The optimised MSFA for the oesophageal dataset, and the resulting classification of the synthetic hypercube are shown in the second and third column, respectively. The optimised MSFA for the colon dataset, and the resulting classification of the synthetic hypercube are shown in the fourth and fifth column, respectively.

No of Bands	Oesophageal MSFA	Oesophageal Classification	Colon MSFA	Colon Classification
**3**	545 (16) 565 (26)<br>601 (20) 601 (20)	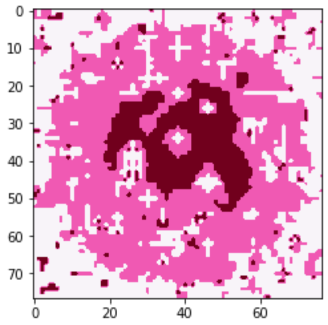	603 (20) 685 (20)<br>555 (20) 685 (20)	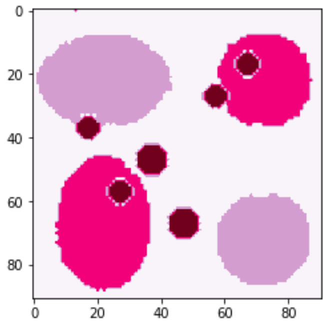
**4**	579 (16) 607 (18)<br>541 (26) 479 (18)	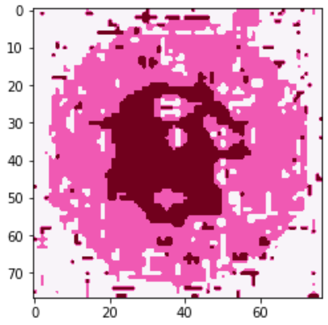	605 (20) 555 (20)<br>655 (20) 685 (20)	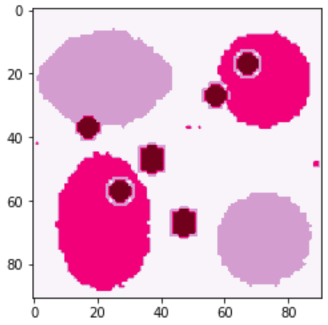

5

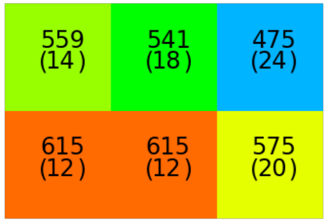
559 (14)
541 (18)
475 (24)
615 (12)
615 (12)
575 (20)

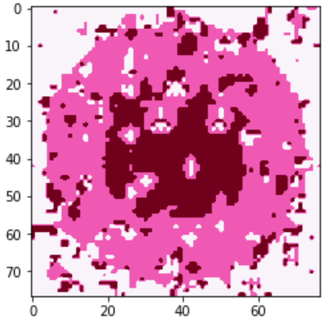
0 10 20 30 40 50 60 70
0 20 40 60

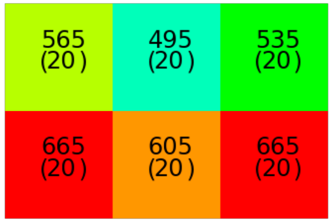
565 (20)
495 (20)
535 (20)
665 (20)
605 (20)
665 (20)

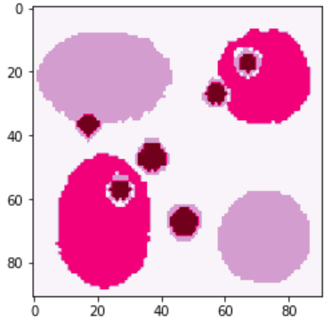
0 20 40 60 80
0 20 40 60 80

6

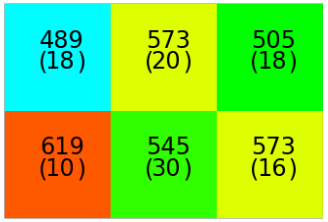
489 (18)
573 (20)
505 (18)
619 (10)
545 (30)
573 (16)

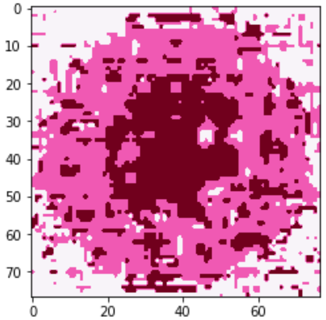
0 10 20 30 40 50 60 70
0 20 40 60

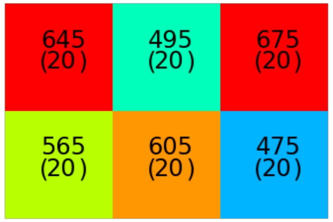
645 (20)
495 (20)
675 (20)
565 (20)
605 (20)
475 (20)

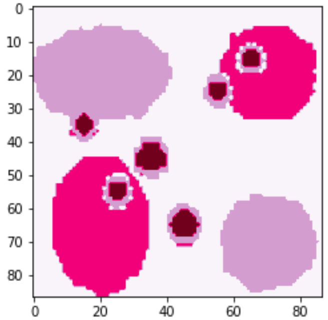
0 10 20 30 40 50 60 70 80
0 20 40 60 80

7

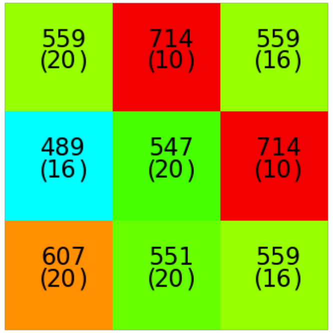
559 (20)
714 (10)
559 (16)
489 (16)
547 (20)
714 (10)
607 (20)
551 (20)
559 (16)

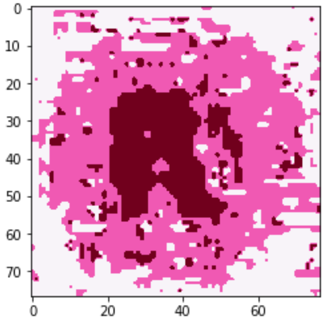
0 10 20 30 40 50 60 70
0 20 40 60

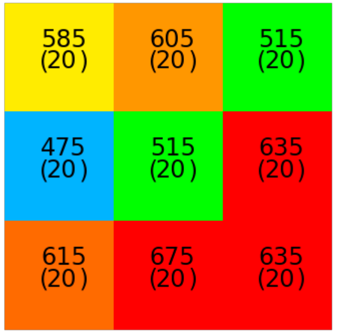
585 (20)
605 (20)
515 (20)
475 (20)
515 (20)
635 (20)
615 (20)
675 (20)
635 (20)

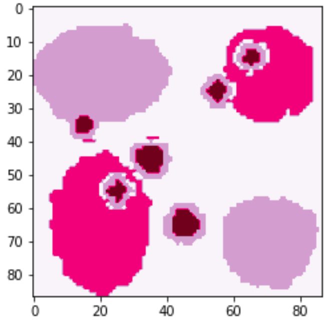
0 10 20 30 40 50 60 70 80
0 20 40 60 80

**8**	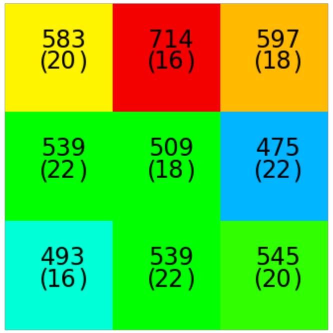583 (20)<br>714 (16)<br>597 (18)<br>539 (22)<br>509 (18)<br>475 (22)<br>493 (16)<br>539 (22)<br>545 (20)	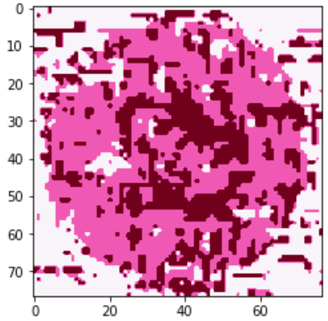	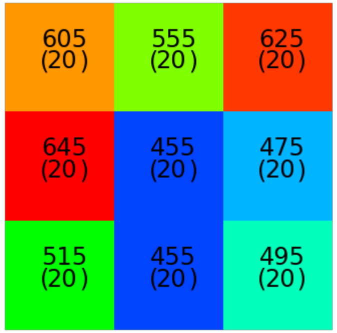605 (20)<br>555 (20)<br>625 (20)<br>645 (20)<br>455 (20)<br>475 (20)<br>515 (20)<br>455 (20)<br>495 (20)	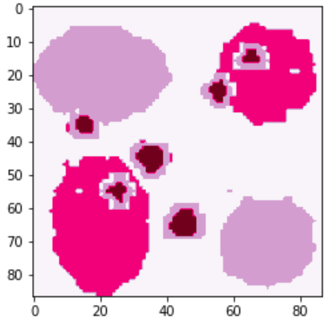
**9**	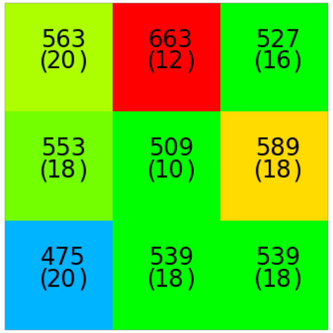563 (20)<br>663 (12)<br>527 (16)<br>553 (18)<br>509 (10)<br>589 (18)<br>475 (20)<br>539 (18)<br>539 (18)	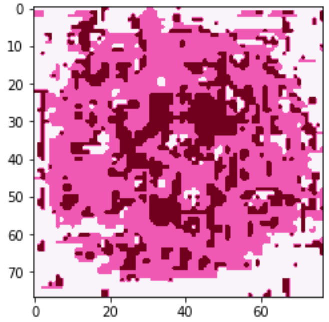	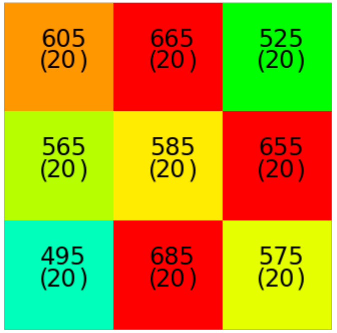605 (20)<br>665 (20)<br>525 (20)<br>565 (20)<br>585 (20)<br>655 (20)<br>495 (20)<br>685 (20)<br>575 (20)	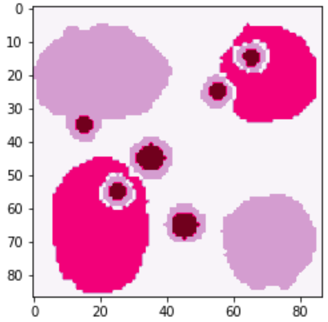

Supplementary Table 3: **SAM Classification of oesophageal and colon data set.** The optimised MSFA for the oesophageal dataset, and the resulting classification of the synthetic hypercube are shown in the second and third column, respectively. The optimised MSFA for the colon dataset, and the resulting classification of the synthetic hypercube are shown in the fourth and fifth column, respectively.

No of Bands	Oesophageal MSFA	Oesophageal Classification	Colon MSFA	Colon Classification
**3**	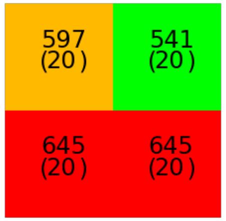	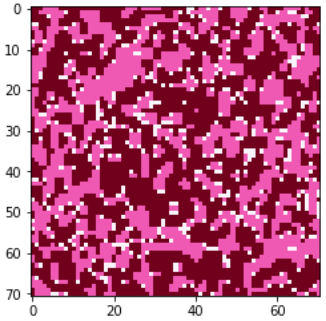	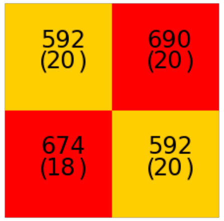	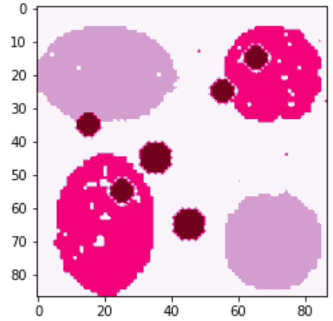
**4**	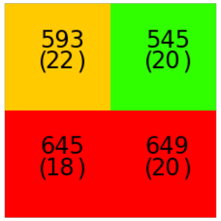	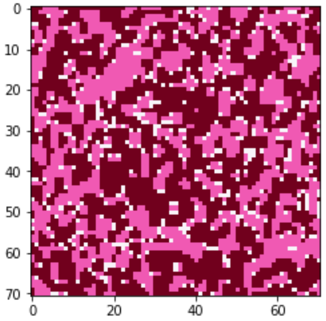	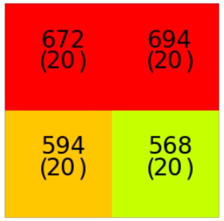	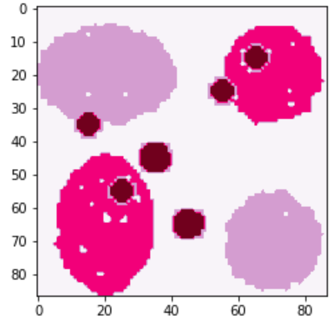

5

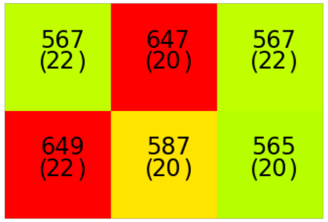
567 (22)
647 (20)
567 (22)
649 (22)
587 (20)
565 (20)

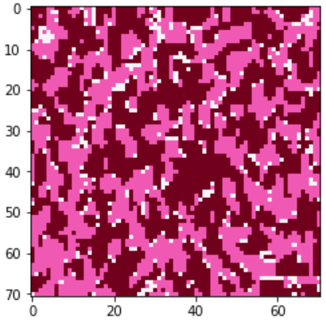

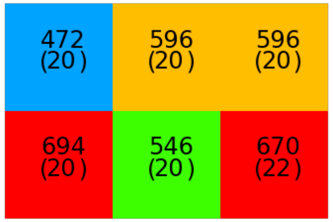
472 (20)
596 (20)
596 (20)
694 (20)
546 (20)
670 (22)

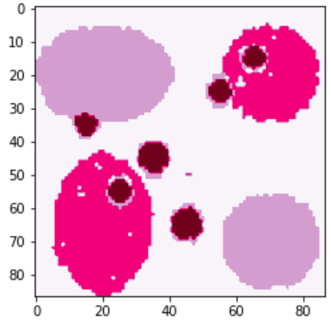

6

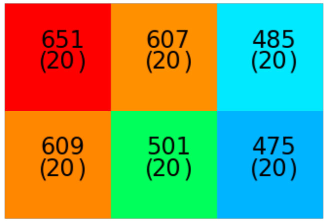
651 (20)
607 (20)
485 (20)
609 (20)
501 (20)
475 (20)

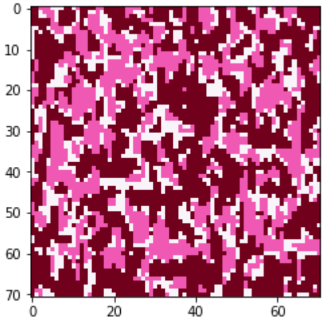

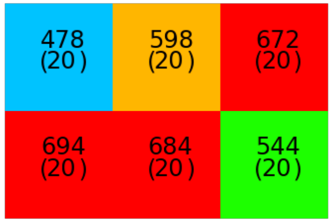
478 (20)
598 (20)
672 (20)
694 (20)
684 (20)
544 (20)

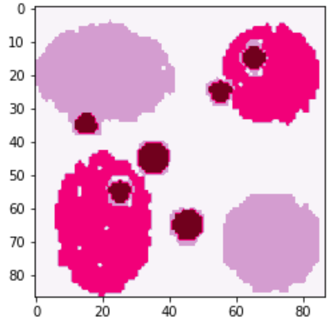

7

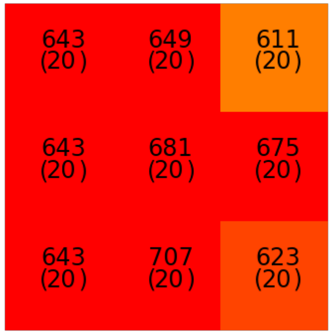
643 (20)
649 (20)
611 (20)
643 (20)
681 (20)
675 (20)
643 (20)
707 (20)
623 (20)

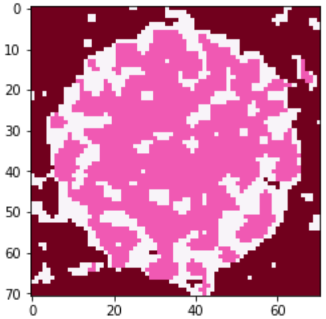

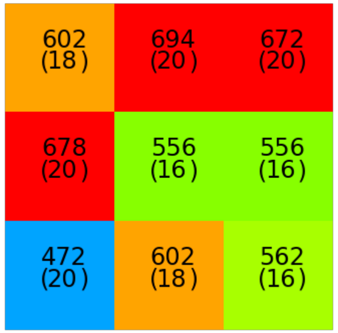
602 (18)
694 (20)
672 (20)
678 (20)
556 (16)
556 (16)
472 (20)
602 (18)
562 (16)

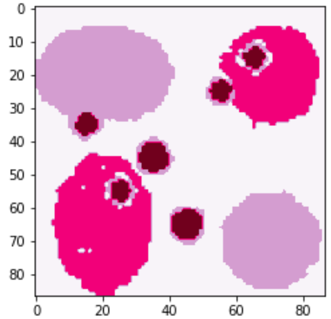

8

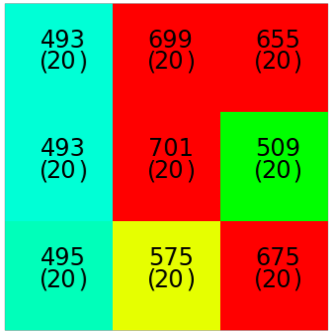
493 (20)
699 (20)
655 (20)
493 (20)
701 (20)
509 (20)
495 (20)
575 (20)
675 (20)

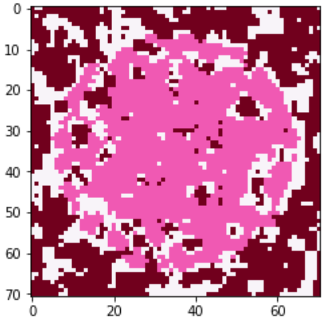

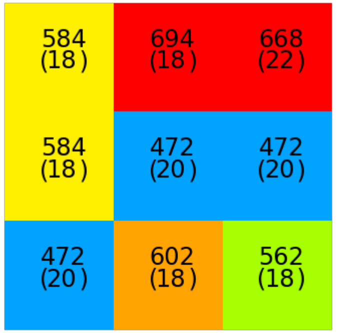
584 (18)
694 (18)
668 (22)
584 (18)
472 (20)
472 (20)
472 (20)
602 (18)
562 (18)

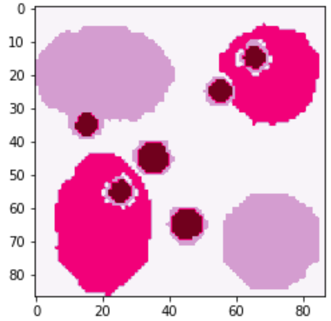

9

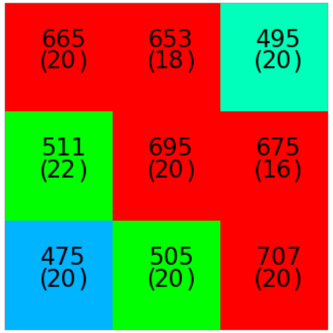
665 (20)
653 (18)
495 (20)
511 (22)
695 (20)
675 (16)
475 (20)
505 (20)
707 (20)

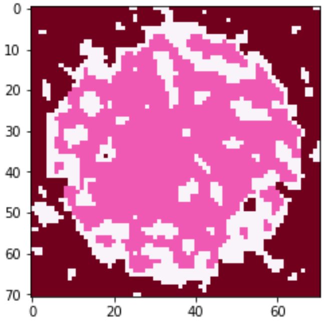

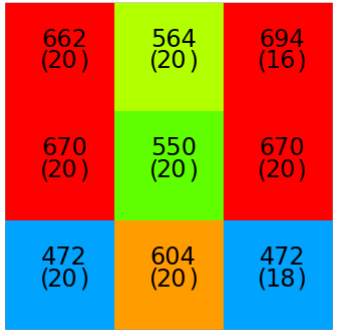
662 (20)
564 (20)
694 (16)
670 (20)
550 (20)
670 (20)
472 (20)
604 (20)
472 (18)

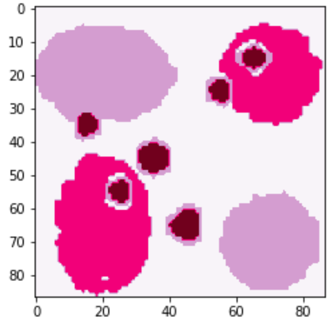

Supplementary Table 4: Normal tissue Best and worst fit calculated values: r^2, HbO_2, Hb, water, scattering, and offset for normal tissue.

	r^2	HbO_2	Hb	Water	Scattering	Offset
Best fit	0.9886	1.6863	0.4970	14.9042	20.8456	16.9042
Worst fit	0.8184	1.4141	0.6235	11.4896	14.4088	17.8684

Supplementary Table 5: Polyp tissue Best and worst fit calculated values: r^2, HbO_2, Hb, water, scattering, and offset for polyp tissue.

	r^2	HbO_2	Hb	Water	Scattering	Offset
Best fit	0.9857	1.5593	0.2969	12.9823	13.7589	17.8368
Worst fit	0.5415	0.5091	0.9167	36.7926	21.5530	16.1552

Supplementary Table 6: Post Resection Tissue. Best and worst fit calculated values r^2, HbO_2, Hb, water, scattering, and offset for post-resection tissue unmixing.

	r^2	HbO_2	Hb	Water	Scattering	Offset
Best fit	0.9902	1.4098	1.2150	-0.2093	6.8400	19.1881
Worst fit	0.4763	1.3215	-0.9968	-46.5925	-25.0397	25.1510

Supplementary Table 7: Hb and HbO_2 unmixing of the colon data set. The optimised MSFA for the colon dataset and the resulting estimates of sO_2 for the synthetic hypercube are shown in the second and third columns, respectively.

<table>
<tr><th>No of Bands</th><th>Optimised MSFA based on unmixing</th><th>sO_2</th></tr>
<tr><td>3</td><td>562 (14) 574 (12)
586 (30) 586 (30)</td><td>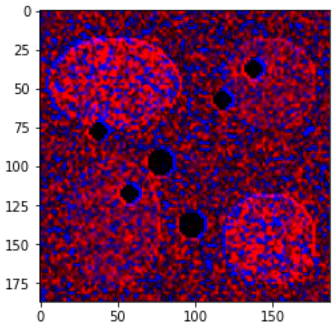</td></tr>
<tr><td>4</td><td>618 (30) 558 (30)
510 (10) 558 (18)</td><td>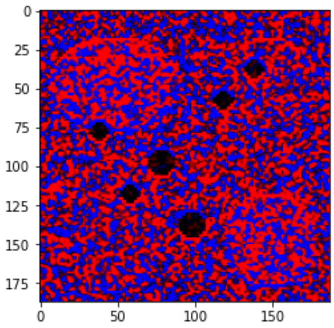</td></tr>
<tr><td>5</td><td>564 (14) 492 (10) 602 (18)
576 (10) 576 (10) 630 (12)</td><td>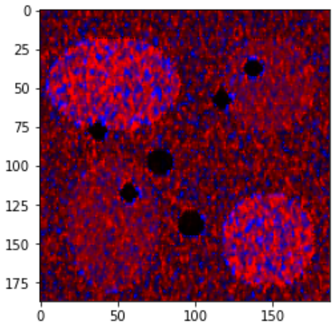</td></tr>
<tr><td>6</td><td>560 (16) 496 (10) 600 (22)
540 (12) 578 (16) 630 (16)</td><td>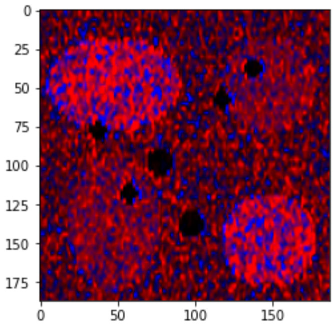</td></tr>
</table>

**7**	494 (16) | 562 (22) | 576 (16)<br>600 (18) | 540 (14) | 562 (22)<br>622 (12) | 514 (24) | 576 (16)	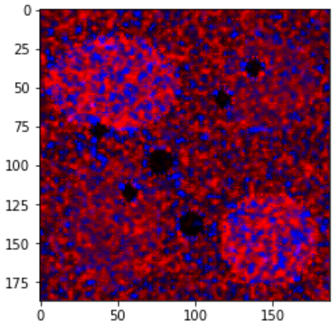
**8**	540 (14) | 556 (30) | 492 (16)<br>572 (22) | 516 (30) | 604 (16)<br>564 (14) | 572 (22) | 616 (18)	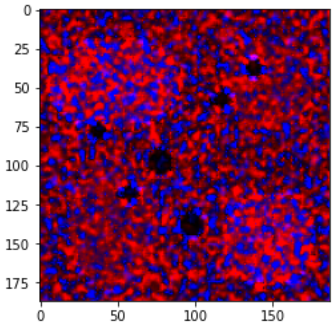
**9**	560 (18) | 540 (26) | 694 (12)<br>572 (18) | 548 (30) | 514 (24)<br>506 (30) | 644 (30) | 490 (16)	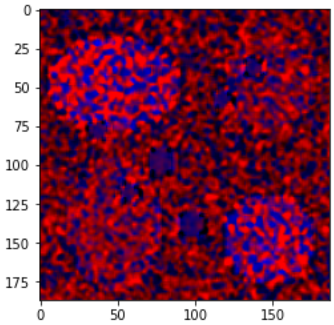
